# Cattle antibodies identify a cross-serotype broadly neutralising foot-and-mouth disease virus epitope

**DOI:** 10.1038/s41541-026-01427-7

**Published:** 2026-04-02

**Authors:** Marie Bonnet-Di Placido, Helen M. E. Duyvesteyn, Angela W. Steyn, Abigail L. Hay, Claudine Porta, Kristel Ramirez Valdez, Elena Lokhman, Sylvia Crossley, Kevan Hanson, William N. Mwangi, Danish Munir, Eva Perez-Martin, Nick J. Knowles, Alison Burman, Abdelaziz A. Yassin, Amin Asfor, Cristina Faralla, Katherine J. Lam, Róisín McComb, Carina Leifeld, Kimberly Pietersz, Donald P. King, Erwin van den Born, Sherie K. Duncan, Bryan Charleston, Elizabeth E. Fry, Jingshan Ren, David I. Stuart, John A. Hammond

**Affiliations:** 1 The Pirbright Institute, Pirbright, Surrey, UK; 2 Division of Structural Biology, The Henry Wellcome Building for Genomic Medicine, University of Oxford, Headington, Oxford, UK; 3 AbCellera Biologics Inc, Vancouver, BC Canada; 4 MSD Animal Health, Boxmeer, AN The Netherlands; 5 Pandemic Sciences Institute, Nuffield Department of Medicine,, University of Oxford, Oxford, UK; 6 CAMS Oxford Institute, The Henry Wellcome Building for Genomic Medicine, University of Oxford, Headington, Oxford, UK

**Keywords:** Immunology, Microbiology

## Abstract

Foot-and-mouth disease virus (FMDV) causes a devastating disease that threatens global food security. Vaccination is hindered by antigenic diversity across serotypes. To identify cross-serotype neutralising epitopes, we isolated 24 FMDV-specific antibodies from cattle sequentially vaccinated with antigens from four serotypes, of which three neutralised three vaccine strains. These three antibodies neutralised 21 and bound 59 additional topotypes across O, A, Asia 1, and C serotypes. Cryo-EM complexes of Fabs with FMD virus-like particles indicated all three recognise a common flexible epitope at the VP1 C-terminus, confirmed by binding competition. Crystallography and structural modelling revealed that a normally inaccessible surface of the hydrophobic VP1 C-terminal peptides inserts into a similar groove in all three antibodies. Comparison of neutralisation activity and integrin receptor blocking by whole antibodies, F(ab’)_2_s, and Fabs suggests neutralisation is mediated by Fc steric hindrance of receptor binding. This cryptic, linear, and cross-serotype neutralising epitope may inform improved FMD vaccines.

## Introduction

Foot-and-mouth disease (FMD) is a highly contagious viral disease affecting cattle, swine, sheep, goats and other cloven-hoofed animals^1–3^. This transboundary disease is economically devastating to livestock farming globally, with a high morbidity and potentially high mortality in younger animals that impacts productivity and trade^4,5^. In endemic countries, or those attempting to eradicate the virus, FMDV is currently controlled by vaccination using inactivated serotype-matched FMDV^6,7^. The protection achieved is short-lived, requiring repeated vaccine boosts to maintain neutralising antigen-specific antibody responses^8–11^. Additionally, the considerable antigenic heterogeneity between and within FMDV serotypes poses significant challenges in producing an effective cross-protective vaccine^9,12,13^. FMDV exists as seven genetically and antigenically distinct serotypes; O, A, Asia 1, C, and the Southern African Territories (SAT) 1–3, type C being extinct^13–15^. Even within a serotype, FMDV topotypes may be differentially neutralised^16–18^.

FMDV is an Aphthovirus of the family *Picornaviridae*. The genome is enclosed in a protein capsid containing 60 copies of each of the four viral structural proteins (VP1-4). While VP4 is internal, VPs 1–3 are exposed externally and form the antigenic surface^19–21^. In the last two decades, target epitopes for protective antibodies on the virus surface have been extensively studied^21–27^. Historically, a linear epitope at the VP1 G-H loop, the site of integrin receptor engagement, was found to be immunodominant in mice^28^, although immunisation with peptide vaccines corresponding to this region did not provide protection in cattle^29^. Other neutralising epitopes have been determined in mice via various methods, leading to the identification of four major antigenic sites (five for the O-serotype)^24,30,31^.

To increase vaccine cross-reactivity, a better understanding of the serotype-specific and cross-serotype epitopes driven by vaccination is needed. For some time, only one structurally determined epitope had been reported^32^ (mouse monoclonal SD6 Fab with C-serotype, 1QGC) but, in the past few years, several cryo-electron microscopy (cryo-EM) Fab/nanobody-FMDV structures have been published^3,6,9,33–37^. Cross-neutralising epitopes appear to exist but rarely drive a dominant antibody response; bovine antibody R55 engages with the VP2 and VP3 loops but despite cross-reacting with two A vaccine strains, A/AF/2 and A/WH/09, only neutralises the latter, due to a single mutation in VP3^9^. The VP1 C terminus (residues 200–213) was initially reported to induce neutralising antibodies after vaccination in mice^38^ and enhance protection in guinea pigs when used as a fusion peptide (FMDV15) with the immunodominant G-H-loop^39^. Further studies using FMDV15 in natural FMDV hosts showed that protection outcomes were mixed^39–43^. Of note, the FMDV15 peptide was shown to induce cross-protection between O, A, and C serotypes, with the VP1 C terminus component deemed responsible, however no neutralisation was reported and the precise epitope was never resolved^41,44^.

Sequential immunisation with multiple FMDV serotypes has shown that a single mixed serotype antigen immunisation can elicit serum cross-reactivity. However, how this translates to individual cross-reactive antibodies remains unclear. Grant et al.^45^ demonstrated clearly that serial heterologous vaccination drives the maturation of cross-reactive B cells, after the first heterologous boost. In addition, this serial vaccination regime maintains high VNT months after the last vaccination with the O antigen when otherwise it has been shown that VNT steadily declines shortly after the booster dose^46^. Where a cross-reactive neutralising epitope was identified using cattle antibody isolation, it required exposure to multiple FMDV serotypes^47^ to identify a single epitope shared between O and A serotypes^9,33^. Even sequential immunisation of llamas with three different FMDV serotypes failed to yield a cross-serotype specific neutralising antibody candidate^48^. Studies and structures of complexes of FMDV with nanobodies M170 and M8^34^ or single-chain variable fragments: F145, B77, C4, R55, R50^6,9,35^ have been O/A-serotype centric, with diverse epitopes identified, which cover most major surface loops, but do not cross-neutralise (except R50, which cross-neutralises O and A). This more recent shift from murine antibodies towards the characterisation of antibodies derived from natural FMDV hosts should provide a more relevant representation of the antigenic landscape.

We report here the isolation of FMDV-specific B cells at peaks of cross-reactivity, obtained by driving a cross-reactive response through heterologous serial immunisation with successful vaccine strains from O, A, and Asia 1 serotypes (based on a previous study^45^). In total, 143 antibody sequences were obtained of which 50 were expressed as recombinant cattle IgG1 antibodies; of the 24 that recognised intact virus-like particles (VLPs), 15 were single-serotype specific, six bi-serotype specific, and three tri-serotype specific. Functional and structural characterisation of the three tri-serotype specific antibodies revealed that they all are neutralising and bind a very similar linear epitope close to the VP1 C-terminus. Further analyses revealed that the mode of action likely involves steric hindrance, with the Fc portion of the antibodies obstructing viral entry. This mechanism highlights the dual importance of epitope recognition and antibody architecture in driving cross-serotype neutralisation and underscores the potential of leveraging such mechanisms for improved vaccine design, particularly as recombinant FMD VLPs can be used as safe and potent vaccines^49^.

## Results

### Heterologous serial vaccination induces and maintains FMDV-specific antibodies

**Fig. 1:**
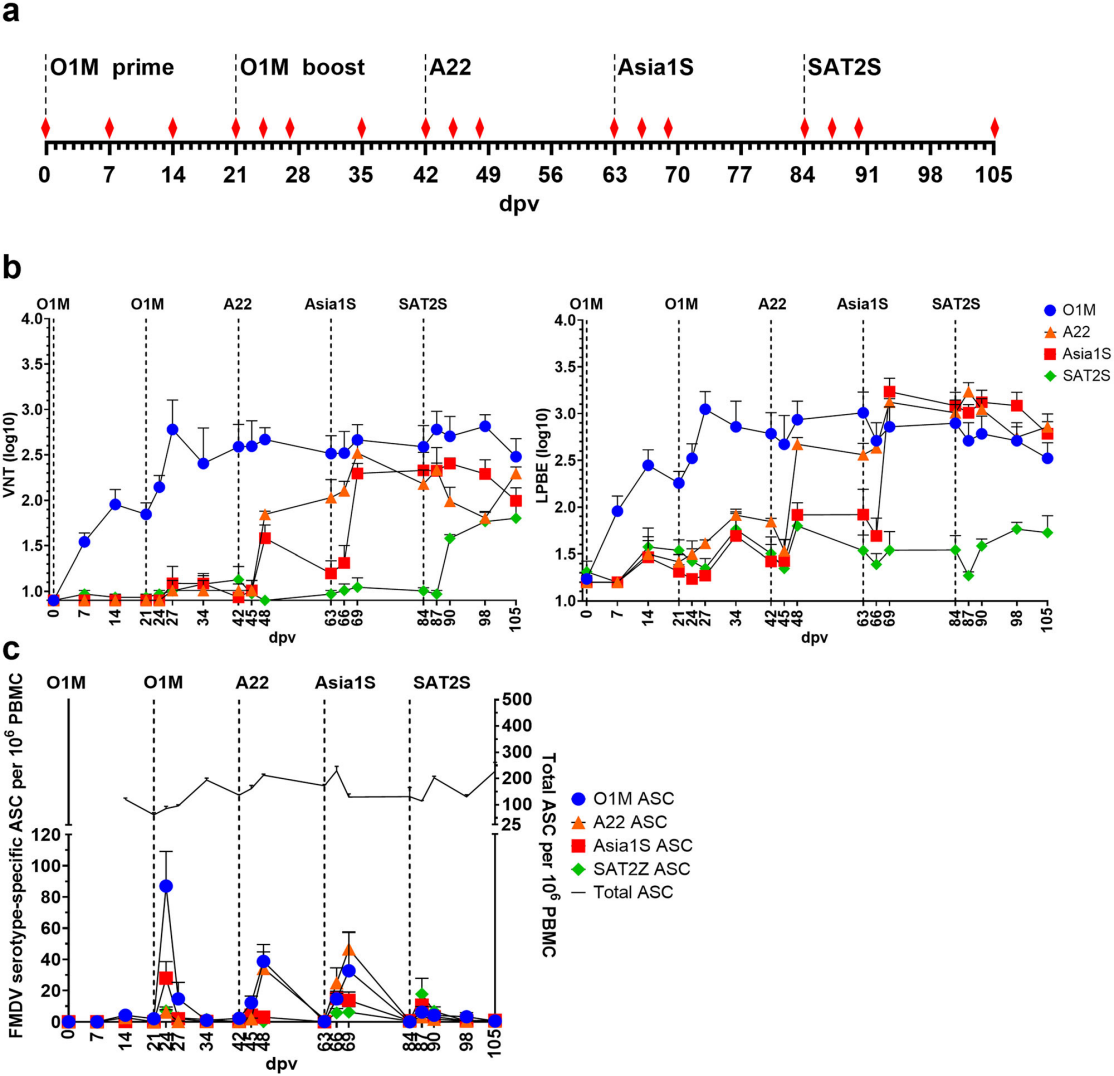
Cattle vaccination regime and outputs. **a** Schematic representation of the cattle vaccination regime. Timeline of vaccinations from day 0 (prime vaccination) until day 105 (end point) post prime vaccination (dpv) is shown. Vaccines were administered every 21 days with the following antigens: prime and boost with O1M, followed by heterologous boosts with A22, Asia1S and SAT2S antigens (dotted lines). Blood sampling time points are indicated by red diamonds. **b** Sera VNT and LPBE titres during the sequential heterologous vaccination timeline. Average titres are shown for viruses of O1M, A22, Asia1S and SAT2S serotypes for each sampling day during the 105 dpv study. Results are expressed as the group mean (4 animals assessed in duplicate). Standard errors of the mean are shown. Vaccination timepoints are indicated by dotted lines. **c** Kinetics of FMDV-specific and total IgG secreting cells in PBMCs during the sequential heterologous vaccination study. Average numbers of serotype-specific antibody secreting B cells (ASC) are shown per 10^6^ PBMC, for O1M, A22, Asia1S, and SAT2Z serotypes VLPs at each sampling day along the 105 dpv. Total IgG ASCs are also indicated in purple. Results are expressed as the group mean (4 animals), and the SEM of the group from duplicate determinations for each animal are shown. Vaccination time points are indicated as a reference (dotted lines).

Four Holstein-Friesian calves were serially vaccinated with FMDV antigens from four distinct serotypes: O_1_/Manisa/TUR/69 (O1M), A_22_/IRQ/24/64 (A22), Asia1/Shamir/ISR/89 (Asia1S) and SAT2/SAU/6/2000 (SAT2S). Following the homologous prime and boost vaccinations with double purified inactivated FMDV O1M vaccine-strain antigens, the calves received three further sequential heterologous FMDV immunisations at 21-day intervals with double purified A22 and Asia1S inactivated vaccine-strain antigens, and with unpurified SAT2S VLPs produced *via* the baculovirus expression vector system^50^ (Fig. 1a) because of the poor stability of the inactivated virus.

Liquid-phase blocking ELISA (LPBE) and virus neutralisation tests (VNTs) were performed to assess the levels of circulating antigen-specific and neutralising antibodies respectively, induced throughout the immunisation regime (Fig. 1b). Following the prime vaccination with O1M, low LPBE and VNT titres were observed, which increased after the O1M boost as expected from previous studies^45,51^. The subsequent A22 and Asia1S immunisations boosted the LPBE and VNT titres against the immunising serotypes but also against heterologous serotypes from the previous immunisations, which remained elevated throughout the study. In contrast, the final SAT2S immunisation at day 84 only slightly increased homologous and heterologous LPBE and VNT titres (Fig. 1b). Overall, LPBE and VNT titres followed similar patterns, and the antigen-specific and neutralising circulating antibodies were boosted by the homologous vaccination and subsequent heterologous vaccinations, except for SAT2S. It is likely that SAT2S failed to induce a strong antibody response on account of using sub-optimally stabilised VLPs which resulted in a lower effective vaccine dose, particularly given that SAT serotypes are known to exhibit reduced capsid stability^52^. Subsequent studies have shown alternative stabilising mutations are required to produce SAT2 VLPs that induce protective immune responses.

### Cross-specific responses are driven from the first homologous boost

Antigen-specific ELISpot assays were used to quantify the number of FMDV-specific antibody-secreting cells (ASCs) within the circulating peripheral blood mononuclear cell (PBMC) population and to assess their cross reactivity. Vaccinia virus-produced VP2 93 C stabilised FMDV VLPs^49^ were used as capture antigens to avoid the detection of any baculovirus-specific B cells that might have been driven by remaining baculovirus in the unpurified SAT2S vaccine preparation. In the absence of vaccinia virus-produced SAT2S VLPs, we used SAT2/ZIM/7/83 (SAT2Z) VLPs, from which VP1 exhibits 74.1% identity with SAT2S (Supplementary Table 1).

Prime vaccination with inactivated FMDV O1M antigen showed very few FMDV-specific O1M ASCs in the peripheral blood at 7, 14, and 21 days post prime vaccination (dpv) (Fig. 1c, Supplementary Table 2). The booster vaccination at 21 dpv with FMDV O1M antigen resulted in a burst of FMDV O1M-specific ASCs and an increase in FMDV Asia1S-specific ASCs at 24 dpv. FMDV A22 and SAT2Z-specific ASCs were also detected at 24 dpv, but at an approximately 5-fold lower magnitude than for Asia1S. Antigen-specific ASCs returned to baseline levels at 34 dpv. The A22 booster vaccination at 42 dpv resulted in a burst of FMDV A22-specific ASCs and in an increase in FMDV O1M-specific ASCs peaking at 48 dpv. The Asia1S boost at 63 dpv induced a small burst in Asia1S- and SAT2Z-specific ASCs at 66 dpv, with higher bursts observed for O1M- and A22-specific ASCs which peaked at 69 dpv. The final vaccination with SAT2S FMDV VLPs at 84 dpv resulted in a smaller burst of ASCs at 87 dpv for O1M, A22, and Asia1S serotypes, and a small increase in SAT2Z-specific ASCs, suggesting a possible suboptimal vaccination with the unpurified SAT2S VLPs. This burst was not detectable by 90 dpv. The O1M prime-boost vaccination induced the highest O1M response three days post-boost, including a notable Asia1S-specific response. In comparison, the A22 and Asia1S heterologous boost maxima were observed at six days post-boost and induced strong cross-specific responses to previous vaccination antigens (Fig. 1c, Supplementary Table 2). The non-vaccinated control (NVC) group had no detectable FMDV-specific ASCs at any of the time points analysed, and the overall ASC response along the study timeline, assessed using total IgG detection, exhibited relatively constant levels for all time points and calves (Fig. 1c).

ELISpots revealed a variable magnitude of responses between individuals, with calf 4 exhibiting a noticeably lower response. However, the best cross-reactive response was observed at the same sampling point in each animal (Supplementary Fig. 1). Also, responses to SAT2S vaccination assessed by LPBE, VNT and ELISpot in all cases were very weak compared to other serotypes (Fig. 1b, c), except for calf 3 (Supplementary Fig. 1). We selected calf 2 at 66 dpv and calf 3 at 69 and 87 dpv for antibody isolation (Supplementary Fig. 1).

### Identification of O1M, A22, and Asia1S cross-neutralising antibodies

**Fig. 2:**
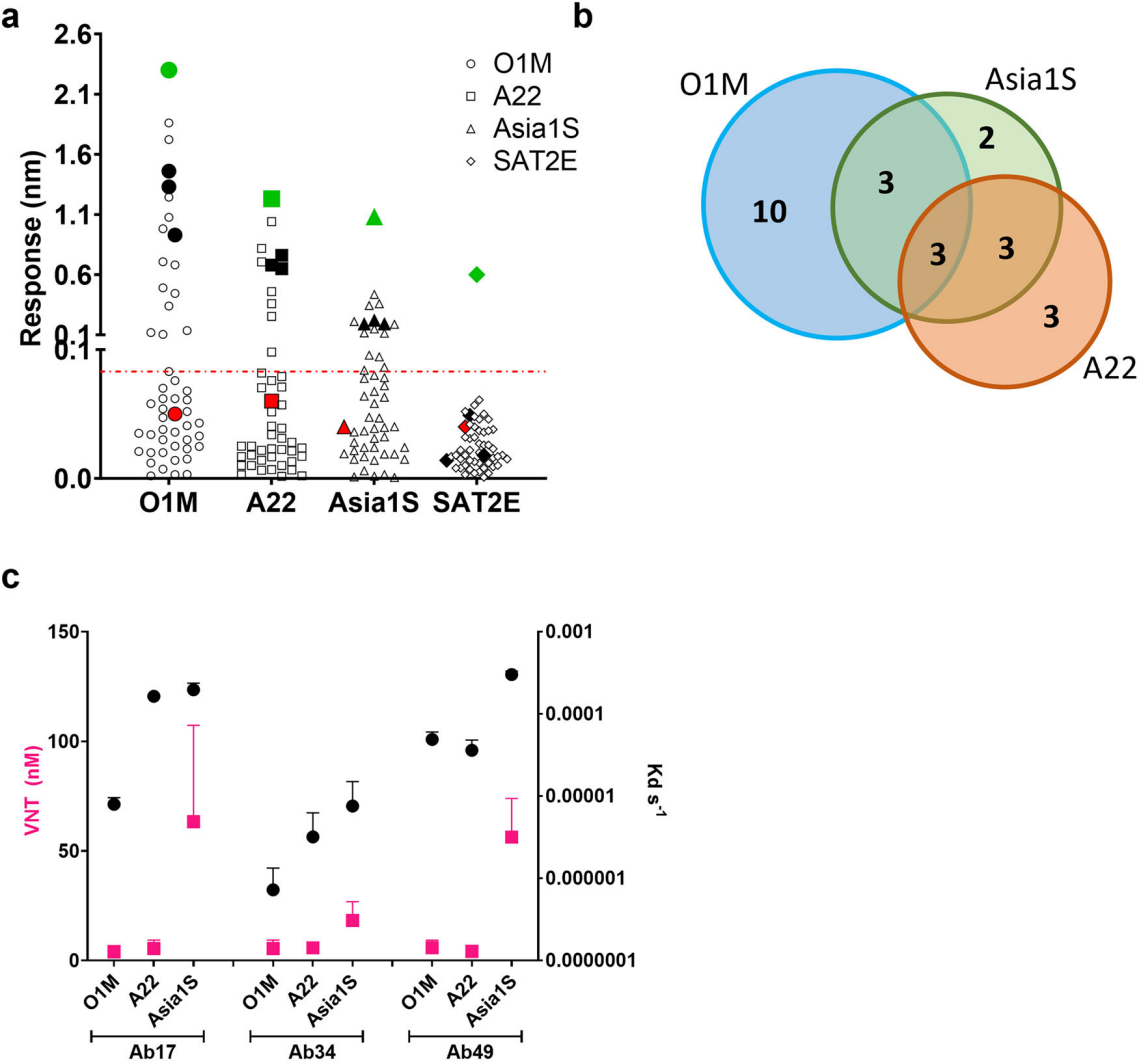
Identification and characterisation of the tri-serotype specific antibodies by biolayer interferometry binding and VNT. **a** Binding response of the 50 recombinantly expressed bovine IgG1 antibodies assessed by BLI. Response values (in nm) to O1M, A22, Asia1S, and SAT2E VLPs are plotted for each antibody. Green symbols indicate positive control samples from serotype-specific sera, and red symbols indicate the responses of B4, an RSV-specific bovine IgG1, as a negative control. Tri-serotype specific antibody responses are shown by solid black symbols. Responses over 0.1 nm indicate binding of the antibodies to the VLP-coated biosensor. **b** Venn diagram showing the relationships between antibodies exhibiting a binding response above 0.1 nm assessed by BLI, for O1M, A22, and Asia1S. Three antibodies were binders to all three FMDV serotypes. **c** Off-rate and neutralisation potency of the three tri-serotype specific antibodies to three FMDV serotypes. Antigen-antibody interactions were measured by BLI (black circles) for O1M, A22, and Asia1S VLPs. Minimum antibody concentrations required to neutralise O1M, A22, and Asia1S viruses were assessed by VNT (pink squares). Values are averages of two (VNT) and three (BLI) independent experiments with standard errors of the mean (SEM) shown.

Antigen-specific IgG-secreting cells were identified using a high-throughput single-cell screening approach that implemented multiplexing of FMD VLP-conjugated beads. A total of 350 B cells with varying specificities identified based on their ability to bind VLPs, but not porcine parvovirus VLPs used as a negative control, were selected and recovered using machine vision and automated robotics-based protocols^53^. From these, 143 unique natural heavy and light chain pair sequences were obtained, of which 50 were selected (based on sequence diversity and heavy chain CDR3 length) to be expressed as recombinant cattle IgG1 antibodies. Biolayer interferometry (BLI) was used to measure VLP-antibody complex formation and avidity at the sensor surface for O1M, A22, Asia1S, and SAT2/ETH/65/2009 (SAT2E), a closer strain to SAT2S than SAT2Z, with 81.5% of VP1 amino acid sequence identity to the former (Supplementary Table 1a). No antibody exhibited a response above the 0.1 nm calculated threshold for binding to SAT2E and 26 antibodies recognised disrupted VLPs. Of the 24 antibodies that bound strongly to at least one serotype, 16 bound O1M, nine bound A22 and 11 bound Asia1S (Fig. 2a, Supplementary Table 3). Overall, 15/24 recognised only one serotype (10 for O1M, three for A22 and two for Asia1S), six bound to two serotypes (three for O1M and Asia1S, three for A22 and Asia1S), and three bound O1M, A22, and Asia1S (Fig. 2b). The latter three, Ab17, Ab34, and Ab49, we term tri-serotype specific antibodies (Fig. 2a).

Focusing on the three tri-serotype specific antibodies, Ab34 displayed the highest avidity for all three serotypes, followed by Ab17 and Ab49 (Supplementary Table 4, Supplementary Fig. 2). The response for Ab34 was: 1.39 nm for O1M, 0.57 nm for A22, and 0.22 nm for Asia1S, with Ab34-VLP association and dissociation kinetics showing strongest avidity for O1M, then A22, and Asia1S in decreasing order (Supplementary Table 4). Ab17 and Ab49 showed a similar hierarchy of responses and avidities (Supplementary Table 4). Neutralisation of all three antibodies was more efficient for O1M than for A22, followed by Asia1S, mirroring the O1M > A22>Asia1S avidities observed by BLI (Fig. 2c).

Of note, these three antibodies are quite distinct from each other. We annotated antibody chain sequences using the Immunoglobulin Multispecies Annotation Tool (IgMAT)^54^. While the light chains of the three antibodies use the same V gene segment (IGLV3-4; Table 1) and are quite similar (approximately 90% variable region amino acid sequence identity between light chain pairs), the heavy chains are quite dissimilar with different CDR-H3 lengths, V gene segment usage, and amino acid sequences (67–70% variable region amino acid sequence identity between heavy chain pairs; Table 1).

**Table 1:**
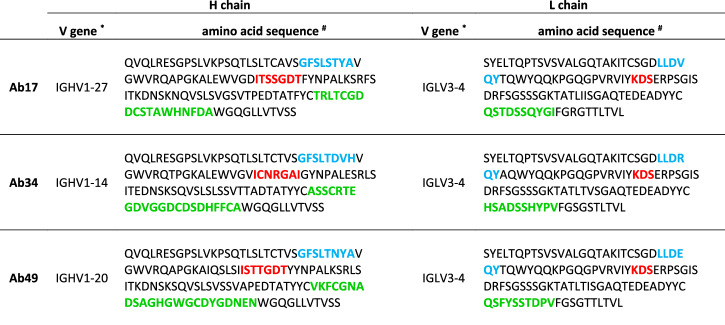
Tri-serotype specific antibody heavy and light chain amino acid sequences

^*^V gene segment usage is annotated for each chain and antibody.

^#^CDR1, CDR2, and CDR3 sequences are shown in bold blue, red, and green, respectively.

### The three tri-serotype specific antibodies are broadly neutralising

**Fig. 3:**
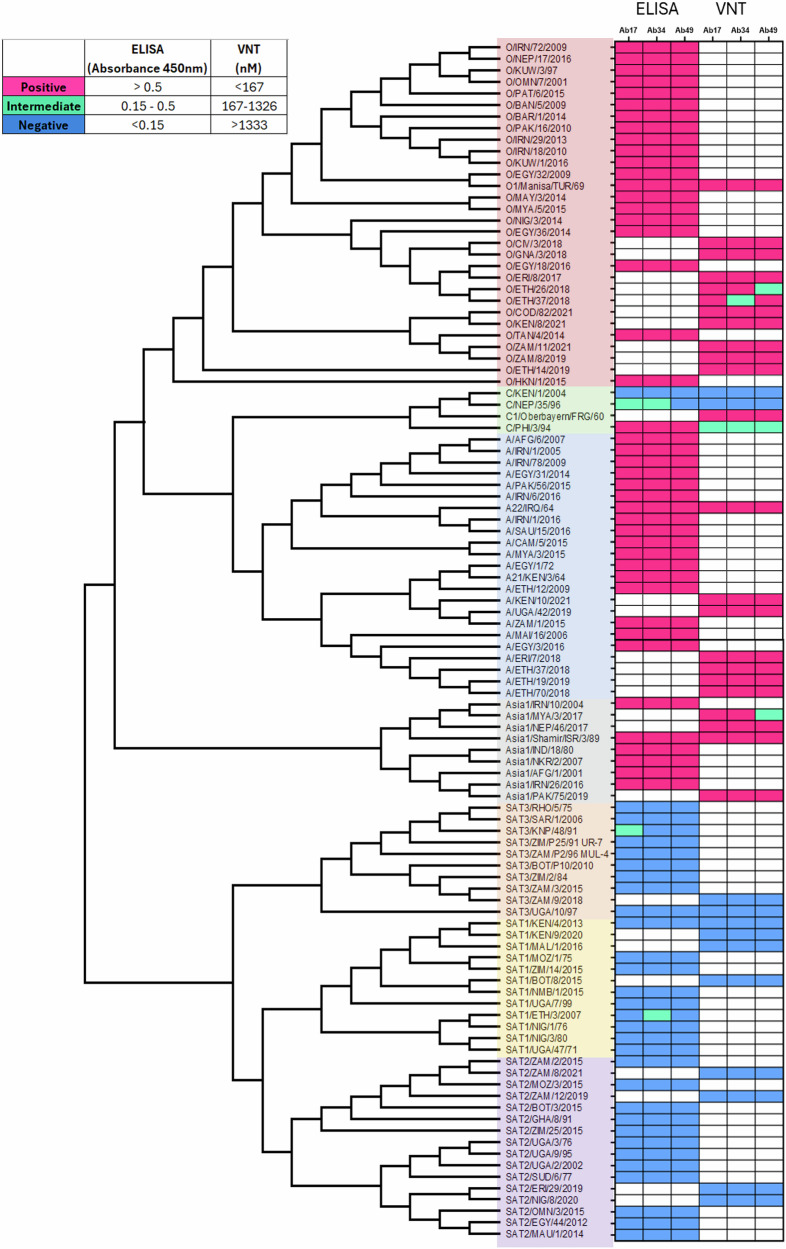
Broad topotype coverage of tri-serotype specific antibodies assessed by ELISA and VNT. Phylogenetic tree of FMDV topotypes tested by ELISA and/or VNT for binding to/neutralisation by Ab17, Ab34, and Ab49. Effect was categorised as positive (pink), negative (blue), or intermediate (green) based on ELISA absorbance at 450 nm or on the minimum antibody concentration (nM) required for virus neutralisation. FMDV isolates are arranged phylogenetically according to full-length VP1 sequences; samples not tested in either assay are indicated in white.

To determine the breadth of tri-serotype specific antibody binding and neutralisation, we selected viruses representing a diverse range of FMDV topotypes for ELISA (76 strains), and VNT (37 strains) (Supplementary Tables 5 and 6). In ELISA, all 20 O, 17 A, and six Asia1 isolates were recognised by each of the three tri-serotype specific antibodies (Fig. 3, Supplementary Table 7). Of the three analysed C viruses, only one, PHI/3/1994, bound strongly to all three antibodies, whilst a second bound at intermediate levels to two of the three Abs. None of the SAT viruses bound to any of the three antibodies except one SAT3 isolate to Ab17, and one SAT1 isolate to Ab34, both at low levels marginally above the calculated 0.17 nm cutoff. The additional 10 O, six A, and three Asia1 viruses were neutralised by all three antibodies, as were the three strains used in the original serial immunisation, at concentrations between 137 pM and 229 nM (Fig. 3, Supplementary Table 7). None of the antibodies were able to neutralise any topotypes from serotypes SAT1, SAT2, and SAT3 at 1.3 µM, consistent with weak or no binding in BLI (Fig. 3, Supplementary Table 7). Interestingly, two of the four C topotypes were also neutralised by all three antibodies at concentrations below 1.3 µM (C/PHI/3/1994 at 213, 426, and 1146 nM and C/Oberbayern BHK1 at 31, 25, and 97 nM for Ab17, Ab34, and Ab49 respectively, Supplementary Table 7), suggesting that the epitope is likely overlapping for all three antibodies. The hierarchy of neutralisation efficiency between the serotypes was preserved across all the topotypes (Supplementary Table 7).

### The epitopes of the tri-serotype specific antibodies are overlapping

To determine if the matching binding profiles and ranking of serotype avidity arise from overlapping epitopes, we performed BLI competition assays. Ab6 (not predicted to compete for epitope binding with the tri-serotype specific antibodies as it is A22 monospecific) was used as a negative control and, as expected, did not compete when used either to saturate the VLPs or as a secondary antibody (Table 2, Supplementary Fig. 3). In contrast all combinations of the three tri-serotype specific antibodies blocked themselves and the other two (Table 2, Supplementary Fig. 3), indicating epitope competition.

**Table 2:** The three tri-serotype specific antibodies compete to bind overlapping epitopes

		Competing antibody			
		Ab17	Ab34	Ab49	Ab6^a^
**Saturating antibody**	**Ab17**	0.003^b^	0.006^c^	0.006^c^	1.160^d^
	**Ab34**	0.004^c^	0.004^b^	0.003^c^	1.090^d^
	**Ab49**	0.003^c^	0.003^c^	0.003^b^	1.040^d^
	**Ab6**^a^	1.200^d^	1.190^d^	1.170^d^	0.002^b^

^a^Ab6 is an A22 monospecific antibody used as a non-competing antibody control.

^b^Self-self blocking interactions are diagonally top left to bottom right.

^c^Responses below 0.1 nm indicate blocking antibody pairs.

^d^Responses above 0.1 nm indicate non-blocking antibody pairs.

### The tri-serotype specific antibody paratopes share a similar groove

**Fig. 4:**
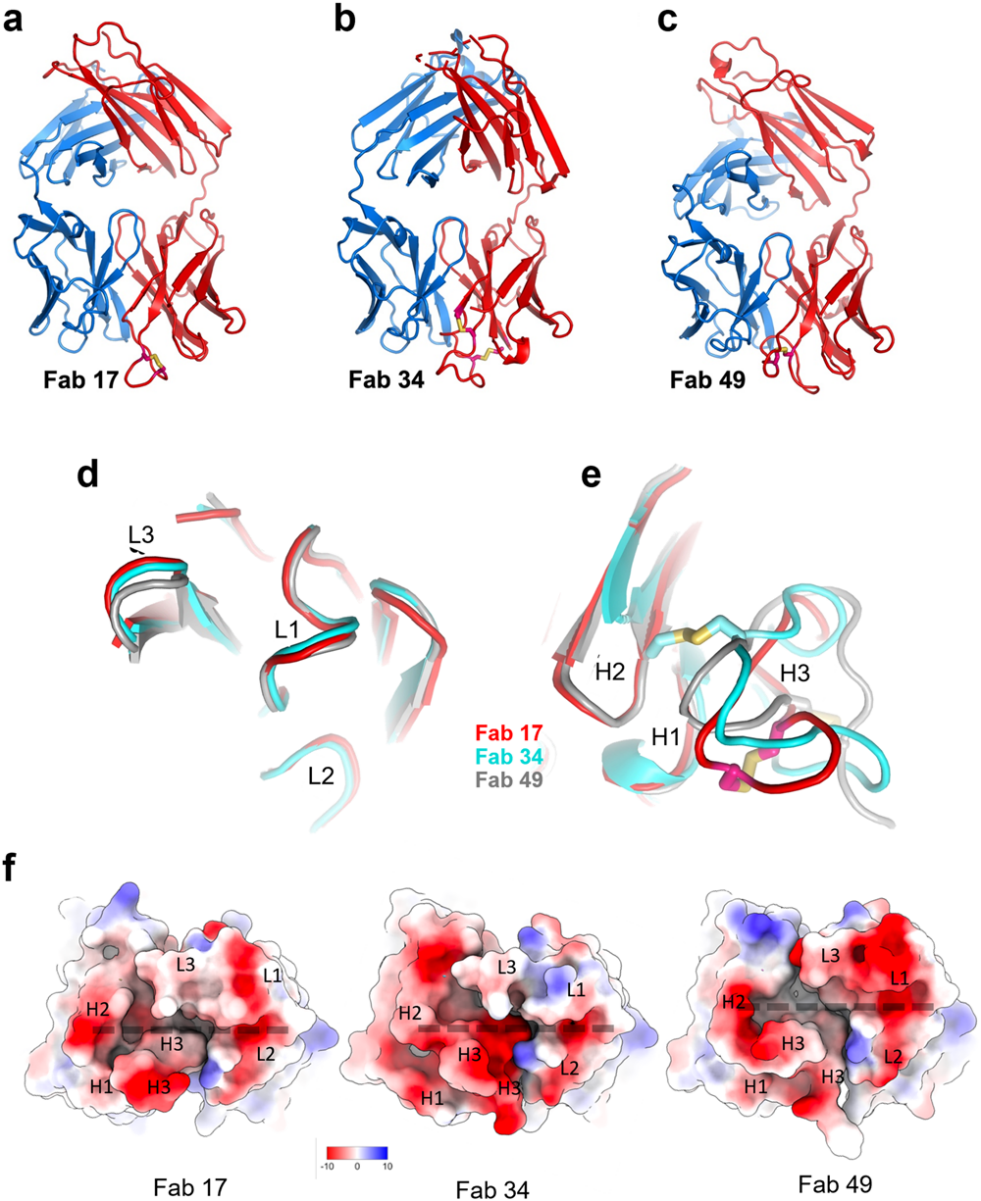
Crystal structures of Fabs of the three tri-serotype specific antibodies. **a–c** Overall structures of Fab 17, Fab 34, and Fab 49, respectively. Heavy chains are coloured in red, light chains in blue. Overlap of the VL (**d**) and VH (**e**) domains of the three Fabs complementarity determining regions L1, L2, L3 and H1, H2, H3 are shown for VL and VH, respectively. Fab 17 is in red, Fab 34 in cyan and Fab 49 in grey. Disulphide bonds in CDR-H3 are shown as sticks in yellow in (**a**–**e**). **f** Views onto the paratopes of Fabs 17, 34 and 49 coloured by electrostatic charge. The common groove is marked by a dashed line.

The Fab portions of Ab17, Ab34, and Ab49 were expressed in mammalian cells, purified, and crystallised. The structures were determined at high resolution (1.72–1.96 Å) at Diamond Light Source (Supplementary Table 8a, Fig. 4a–c). As expected, the light chain variable regions are very similar (mean RMSD for 106 Ca atoms 0.46 Å between the three pairs of structures, Fig. 4d). The lengths of CDR-H1 and CDR-H2 are the same for the three antibodies and they have similar conformations. However, the CDR-H3s vary not only in length (from 18 to 23 residues) but also in disulphide bond positions (5th and 9th, 4th and 21st, and 4th and 16th residues for Ab17, Ab34, and Ab49, respectively; Fig. 4e). There is a third cysteine residue in the CDR-H3 of Ab34, sited between the two disulphide bonded residues, which makes a disulphide bond to CDR-H2 (Fig. 4b). These differences result in paratopes with significant differences, not only in topography, but also in overall charge (Fig. 4f). Despite these differences, inspection of the surfaces reveals that all three bear a groove running between the heavy and light chains, which shares a similar shape and has common charge features including a negative charge at both ends of the groove (Fig. 4f). This groove is suggestive of peptide binding.

### The tri-serotype specific antibodies attach similarly to a common flexible epitope

To determine the binding site of the three cross-neutralising antibodies, cryo-EM analyses of complexes of Fabs with FMDV VLPs were performed. Grids were prepared of O1M VLPs bearing the stabilising VP2 93 C mutation mixed with molar excesses (300:1 ratio) of Fabs. Standard analysis methods yielded icosahedral 3D reconstructions at 2.7, 3.4, and 3.4 Å resolution for Fab17, Fab34, and Fab49, respectively complexed with O1M VLPs (Supplementary Table 8).

**Fig. 5:**
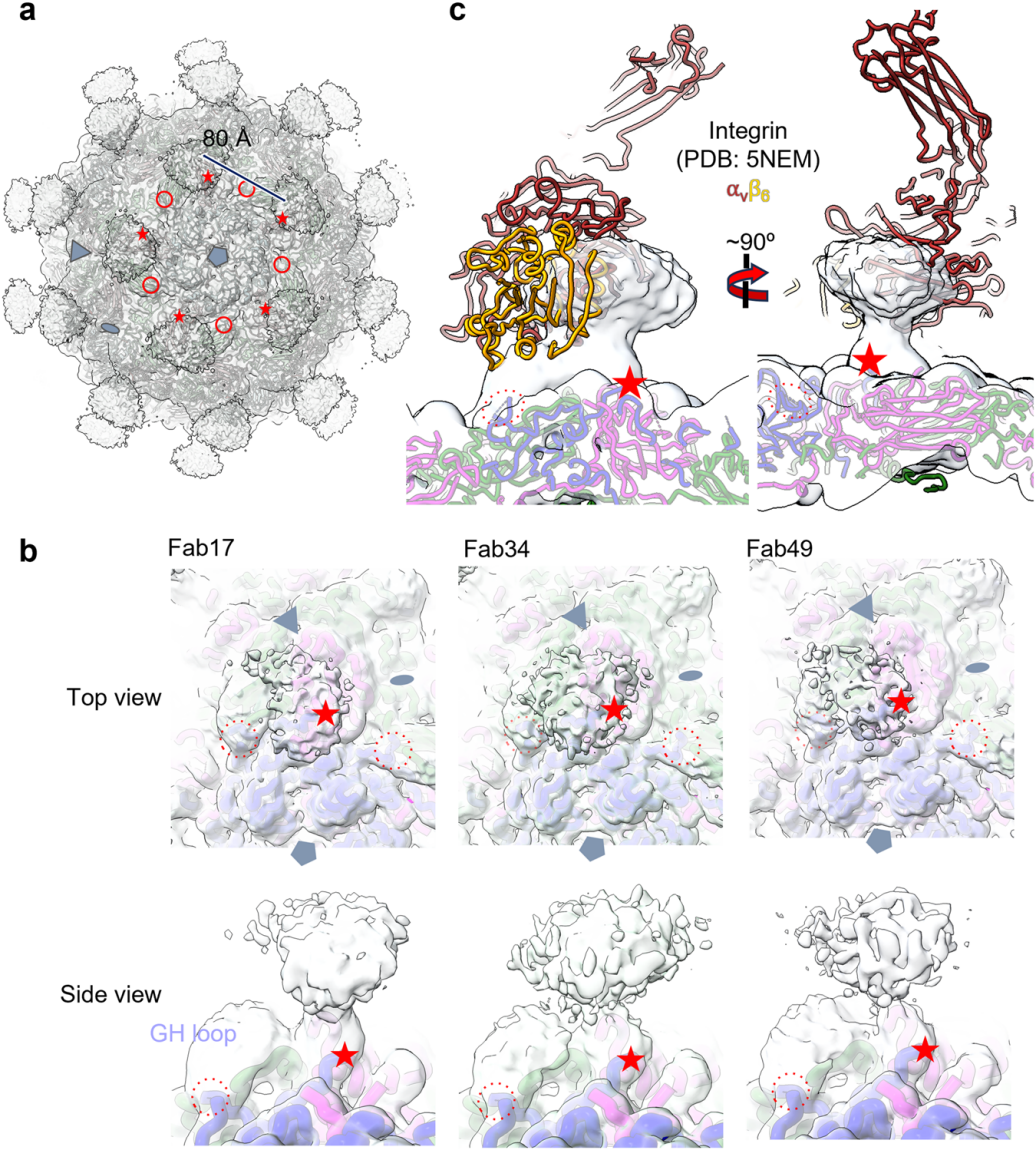
Tri-serotype specific Fab epitope mapping using cryo-EM. **a** Complex of O1M VLPs with Fab34, density with an applied B-factor of 500 Å. For one penton, the VP1 G-H loops (N and C terminal ends) are circled and the VP1 C-termini are indicated by stars. Pentamer, triangle, and oval symbols correspond to the 5-, 3-, and 2-fold axes respectively for one monomeric unit. **b** Globally refined maps of O1M VLPs complexed with each of the tri-serotype specific Fabs with the model shown for one monomer coloured by capsid protein (VP1 blue, VP2 green, VP3 pink). The C-terminus of VP1 is indicated by a star and the position of the G-H loop on adjacent protomers is shown by circles in each panel. **c** Side view of a single Fab density from the B-factor ‘blurred’ cryo-EM map of the Fab34-O1M complex with capsid protein models coloured blue, green, pink for VP1, 2, 3 and overlay of integrin model, shown in red and gold. The C-terminus of VP1 is marked as a star and the VP1 G-H loop termini are circled.

As expected for tight-binding antibodies, decoration of the VLPs was visible in the raw micrographs (Supplementary Fig. 4). Nevertheless, although the reconstructions were derived from large numbers of particles (>10 K in each case), yielding high resolution density for the VLPs, density attributable to Fabs was weak in all reconstructions. Indeed, B-factor blurring to ~500 Å^2^ was required to clearly visualise the density (Fig. 5a). Since the 300:1 molar ratio of Fabs to VLPs would be expected to produce high occupancy binding, this suggests that the Fabs were flexibly attached to the particles. To investigate if several discrete structures were present, symmetry expansion and local refinement was performed^55^. This failed to recover any reliable structures, confirming that attachment was to a flexible epitope. Comparison of the icosahedral reconstructions shows that the three Fabs occupy an overlapping space (Fig. 5b), which also intersects with the integrin-binding site at the RGD motif in the G-H loop (Fig. 5c). Note that the Fab density is strongest for Ab34, the most potent of the three antibodies.

To map the region of interaction, a model of O1M^56^ was rigid body fitted to each of the three reconstructions. This demonstrated that the interactions were centred on the region occupied by the VP1 C-terminus (Fig. 5b). The extreme C-terminus of VP1 (residues 209–213) is disordered in density maps of un-complexed O1M and is further disordered in the present Fab complex structures (the C-terminal 17 residues, 197–213, are invisible in the maps), consistent with attachment of the Fabs causing these residues to be dislocated from the VLP surface (see below).

### A linear epitope in the VP1 C-terminus mediates tri-serotype specific antibody binding

The structural data supporting binding of the tri-serotype specific antibodies to flexible VP1 C-termini and the presence of grooves in the antibody paratopes, led us to assess whether they recognise conformational or linear epitopes. In a western blot with VLPs reduced into linearised protein subunits, all three tri-serotype specific antibodies bound to a 24–25 kDa band, consistent with the VP1 subunit. Mouse anti-VP2 antibody 4A3, a cross-serotype-specific antibody known to bind a linear epitope^57^ also exhibited binding to the three denatured VLPs tested, O1M, A22, and Asia1S (Supplementary Fig. 5). Ab117 (an antibody that binds an internal conformational epitope^33^) and B4 (an irrelevant cattle antibody) did not bind.

**Fig. 6:**
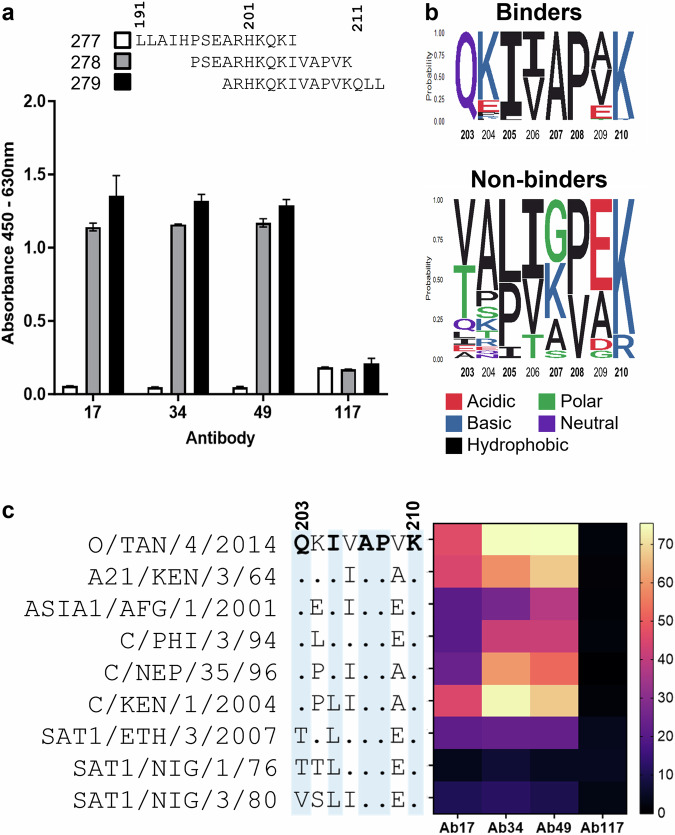
Tri-serotype specific antibodies target a conserved linear epitope at the VP1 C-terminus. **a** ELISA reactivity of Ab17, 34, 49 and negative control Ab117 with O1M peptides designed to broadly cover the VP1 C terminus region. Alignment of these peptides with each other is also shown. **b** Sequence logo indicating the frequency of each amino acid at positions 203–210 of VP1 in the viruses bound (top) and not bound (bottom) by the tri-serotype specific antibodies. **c** Average RUMax for SPR peptide binding with Ab17, 34, and 49. Ab117 was included as a negative control. Each block represents the percentage of RUMax derived from three independent couplings on the same chip. Each line is a peptide from the virus strain indicated, with its sequence shown relative to that of O/Tan used as the reference. Amino acids highlighted in blue are highly conserved across all viruses bound and neutralised by the tri-serotype specific antibodies.

To identify a precise binding motif, three overlapping peptides covering the last 21 amino acids of the C terminus of VP1 from serotype O/UKG/34/2001 were tested for antibody binding by ELISA. Binding was observed to peptides covering residues 196–210 and 199–213, but not 191–205, indicating that the presence of amino acids 206 to 210 is required for interaction (Fig. 6a). Alignment of the VP1 C-termini of binding and non-binding viruses revealed that several residues in this “core” 206–210 region (specifically amino acids 207 (A), 208 (P), and 210 (K)) are conserved across the 64 viruses which are bound by the tri-serotype specific antibodies (Fig. 6b). Two residues outside the “core”, namely 203 (Q) and 205 (I or L to a lesser extent), were also conserved in bound viruses. To further investigate the contribution of this core and surrounding residues, a panel of 13 peptides was designed to encompass binding and non-binding viruses with identical “core” motifs but variable surrounding residues (Fig. 6c). Peptides from the two non-binding C serotype viruses (C/KEN/1/2004 and C/NEP/35/96, Fig. 6c) were able to bind the tri-serotype specific antibodies, albeit not as strongly as the C/PHI/3/94 virus peptide which bound as complete virus. These two C topotypes were the only non-bound viruses to carry the ^203^QxI/LxAPxK^210^ motif (Fig. 6c). This suggests that despite the VP1 C-terminus motif of ^203^QxI/LxAPxK^210^ being sufficient and key for antibody binding, other parts of the capsid structure might, directly or indirectly, impact the antibodies’ ability to bind intact virus.

### The C-terminus of VP1 binds as an extended peptide in a groove on Fab34

**Fig. 7:**
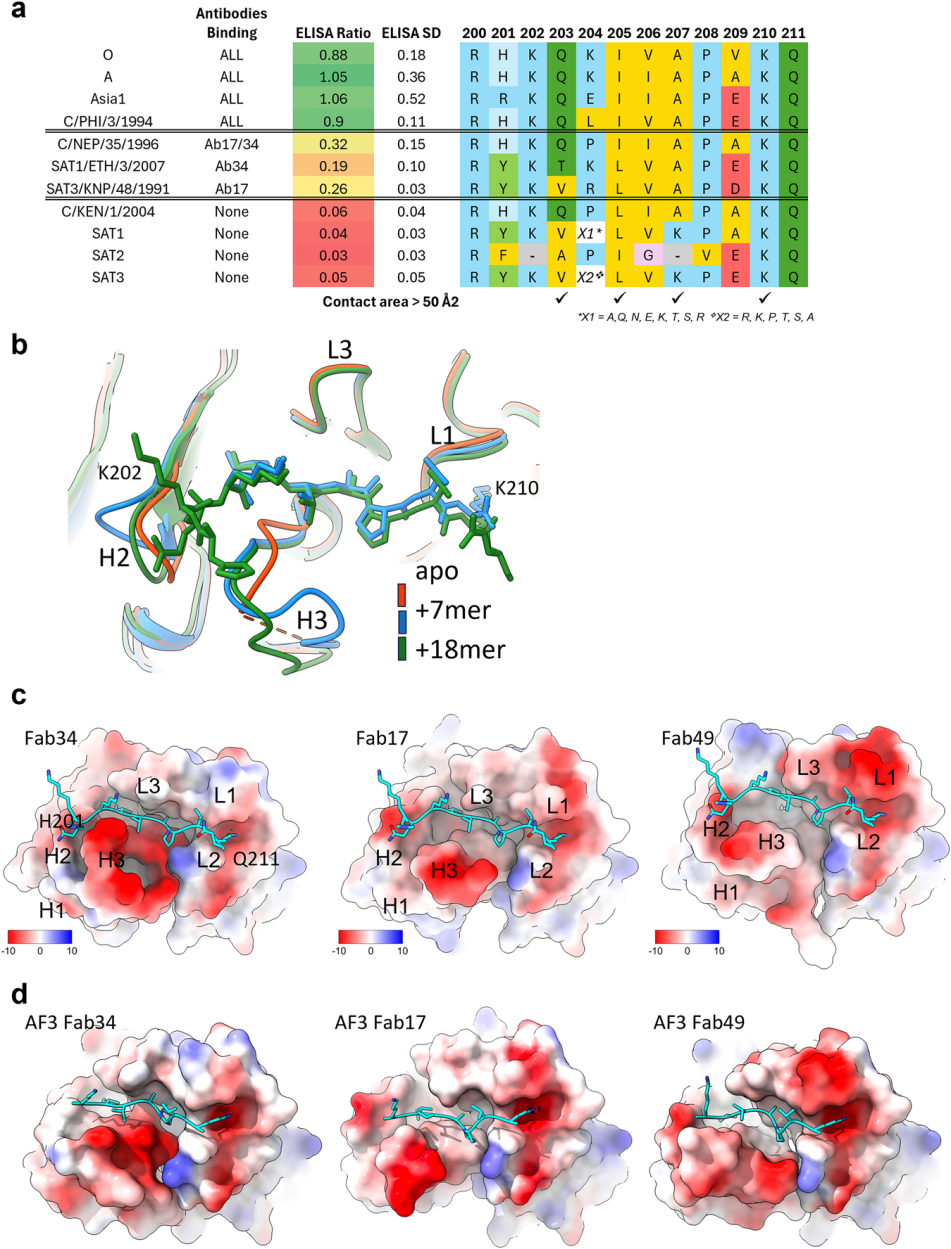
Binding of C-terminal VP1 peptide to the tri-serotype specific Fabs. **a** Summary of ELISA results with a VP1 C-terminus sequence alignment (corresponding to residues 200–211 in O1M) for each of the listed viruses. The rows are grouped by the ELISA signal, which represents the average for the three antibodies across all viruses (except where a specific topotype is indicated in which case the average for the three antibodies for that topotype is shown). Standard deviation (SD) is also shown. For O, A, Asia1 and SAT2 the sequence displayed is the most prevalent within the viruses tested. For SAT1 and SAT3 sequences are more diverse with no single prevalent sequence present. Instead, the most prevalent amino acid for each position is shown and variability at position 204 is highlighted. **b** Overlay of two peptide/Fab34 structures determined by co-crystallisation, the 7-mer peptide complex is in blue, the 18-mer complex in green and the apo Fab model is in orange. **c** The left panel shows the Fab electrostatic surface for the 18-mer peptide/Fab34 complex structure. In the two panels to the right, this peptide structure has been mapped onto the two other Fabs by superimposing the variable domains of the Fabs. Heavy (H) and Light (L) chain CDRs are marked (H1-3, L1-3). **d** Representation as in C, however in this case all coordinates are derived from AF3 predictions using the 7-mer peptide and Fab sequences.

Based on the Fab crystal structures and peptide binding studies by ELISA and SPR, two C-terminal peptides of VP1 from virus O1M (an alignment of residues in this region is shown in Fig. 7a) were synthesised and co-crystallised with Fab34 (chosen as the tightest binder). The shorter 7-mer peptide corresponds to residues 204-210 of the VP1 C-terminus (and includes the core ^203^QxI/LxAPxK^210^ motif defined above (Fig. 6b), while the longer 18-mer peptide corresponds to VP1 residues 194-211. Data were collected from a single crystal for each peptide-Fab34 complex, and the structures were determined to 1.8 and 3.9 Å respectively (Supplementary Table 8a).

In both cases, the peptide is bound, as expected, in the groove between the heavy and light chains (Fig. 7). Whilst all seven residues of the shorter peptide are well ordered, only residues corresponding to C-terminal residues 201 to 211 are visible for the longer peptide; however, the residues shared between the two peptides are bound almost identically (RMSD < 0.2 Å). Whilst the light chain structure is essentially identical between the two peptide-bound structures, the heavy chain shows some differences, notably CDR-H2 and H3, which are close to the three extra ordered residues at the N-terminus of the longer peptide, are rearranged by several Å (Fig. 7b). Interestingly, comparison with the apo Fab34 shows that both loops are in different conformations (Fig. 7b) and are rather flexible (as judged by the high B factors for CDR-H3 and a complete loss of interpretable density for CDR-H2). These changes are by far the most significant differences between the apo and peptide-bound Fab structures.

**Fig. 8:**
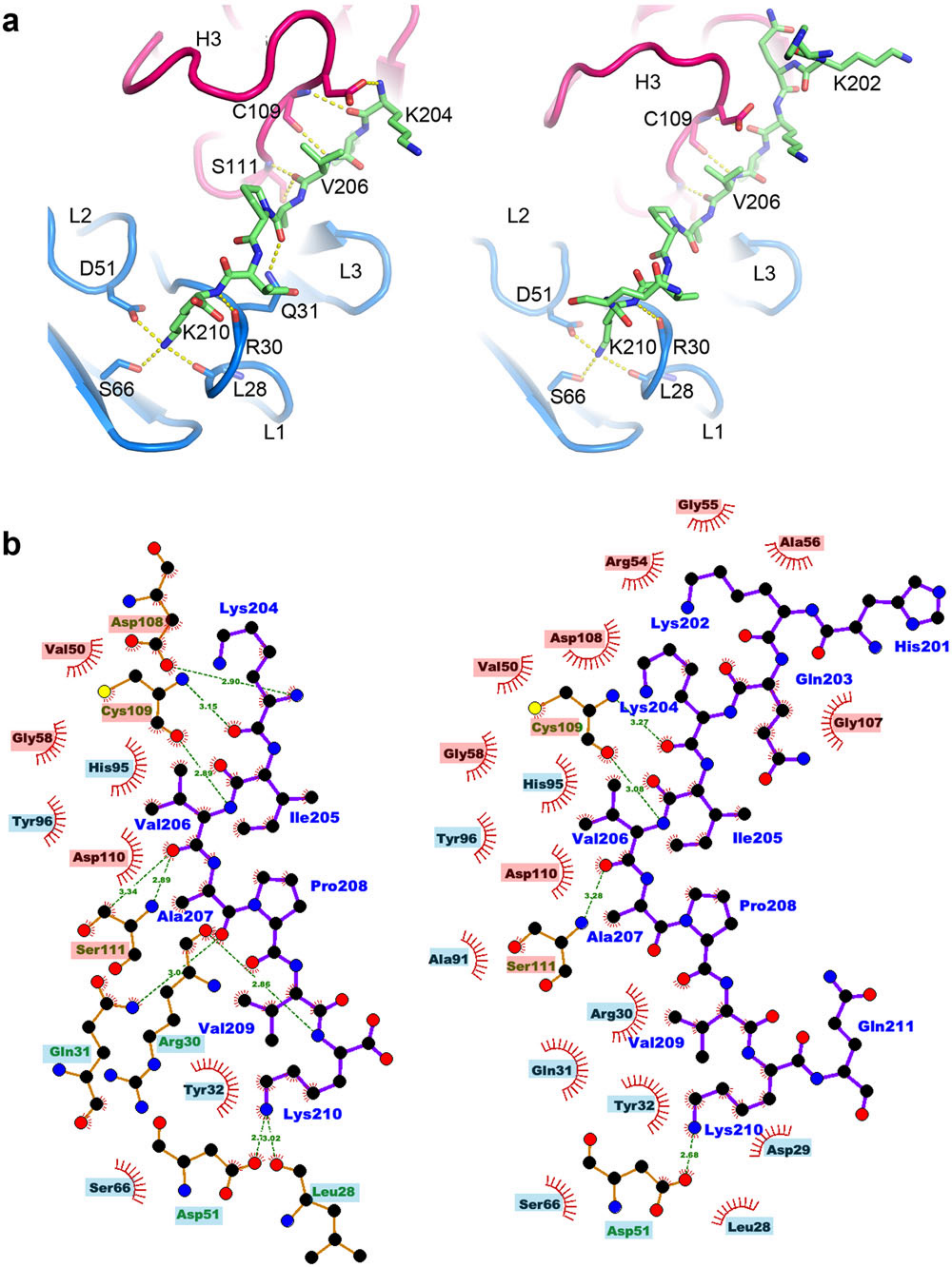
Details of peptide binding interactions with Fab34. **a** Structures for 18-mer (right) and 7-mer (left) peptides viewed from above the peptide binding groove. The light chain is in blue, heavy chain in red, and peptide in green. H-bonds determined in Chimera^88^ are shown as dashes. Heavy and light chain CDRs are labelled (H3, L1-3). **b** Ligplot+^89^ representation of the 7-mer and 18-mer (left and right, respectively) interactions. Heavy chain residue labels are highlighted in red, those of the light chain in blue. Note key H bond interactions between K210 and CDR-L1 D51 and residues V206 and K204 with the C-terminal portion of CDR-H3.

The bound peptide is fully extended in the central region in both the short and long versions and makes extensive contacts with both the heavy and light chains (the 7-residue peptide alone makes ~627 Å^2^ of interface^58^, with similar contact areas between the light and heavy chains). In addition to nine H-bonds and salt bridges, there are numerous hydrophobic interactions. Notably, at the heart of the interaction, the peptide from residues 204–206 forms a β sheet interaction with the C-terminal part of CDR-H3 (Fig. 8), providing a core interaction that is antigen sequence independent.

The 7-residue peptide (K_204_IVAPVK_210_) comprises five hydrophobic residues flanked by basic residues with isoleucine 205 and alanine 207 projecting inwards into pockets between the CDR-H3 and CDR-L3 loops. In addition, the final lysine (K210) projects into a negatively charged pocket. For the 18-mer peptide, the side chain of residue Q203 is sandwiched by CDR-H2 and H3. These four residues (203, 205, 207, 210) project into the groove of the Fab, are key to recognition, and are highly conserved in the serotypes that show binding but less so in the SAT serotypes (Fig. 8). C/NEP which shows weak binding shares these four key residues but unlike C/PHI, which binds well, has P204 which may distort the peptide conformation. SAT1/ETH and SAT3/KNP which show very weak binding, only share A207 and K210. C/Ken, which does not bind, shares three of the residues, has P204 which will weaken binding, but also has isoleucine 205 replaced by a leucine which is presumably poorly accommodated.

### Antibody binding requires disengagement of the VP1 C-terminus from the VLP and conformational rearrangement

Antibody attachment requires a conformational rearrangement of the C-terminus of VP1. Superimposition of the bound peptide of the 7-mer-Fab complex onto the corresponding C-terminal residues in the context of the apo O1M VLP leads to a clash between the bulk of the Fab and the VLP (Supplementary Fig. 6). Whilst this is not entirely surprising, since the protein/protein interaction face of the peptide is used for both interfaces, it does mean that the C-terminal peptide must be peeled off the VLP surface and re-orientated to allow attachment of the Fab.

### A common mode of engagement for all three cross-reactive mAbs

Since all three antibodies bind in a similar way to intact particles and all possess a groove on their binding surface, we investigated whether, despite the differences in the Fab structures, the mode of engagement with the VP1 C-terminus might be similar. As a first check, the peptides from the Fab34/peptide complexes with both the short and the long peptide were superimposed on the Fab17 and Fab49 structures (Fig. 7c shows the 18-mer peptide). In each case, the peptides fitted snugly into the binding groove, without significant clashes, suggesting a similar binding mode of the core peptide. However, we hypothesised that the actual binding may show a slight displacement of the peptide, with the flanking residues of the peptides differing between each other due to the varying surface topologies. To assess this, we first investigated whether Alphafold 3^59^ was able to correctly predict the binding of the short peptide to Fab34 (Fig. 7d). Predictions show the peptide correctly positioned within the Fab groove, with only minor errors in the peptide conformation (Fig. 7d, left panel). Encouraged by this, we ran Alphafold 3 on Fab17 and Fab49. The results (Fig. 7d, centre and right panel) predict very similar binding to all three antibodies.

### The tri-serotype specific antibodies overlap the volume occupied by the integrin receptor when bound to VLPs

Structural and functional studies have demonstrated that the arginine-glycine-aspartic acid (RGD) cell adhesion motif, is recognised by integrins such as αvβ3 and αvβ6 and essential for viral cell entry^60–66^. The RGD motif is located in the highly flexible VP1 G-H loop (residues 133-157 in serotype O), which is proximal to the VP1 C-terminus on the VLP surface and usually disordered. The VP1 C-terminal ‘tails’ wrap around the 5-fold icosahedral axes with the tail of one icosahedral subunit falling close to the G-H loop of the clockwise-related subunit. Having previously visualised the binding of integrin receptors to FMDV VLPs^56^, we mapped the position of bound integrin onto the image reconstructions of the bound Fabs from the three cross-reactive antibodies (Fig. 5c) to understand whether the proximity of the VP1 C-terminus to the G-H loop could impact integrin binding. Most of the three Fab densities fall within the molecular envelope of the primary conformation of bound integrin, as illustrated by the O1M VLP/Fab34 complex (Fig. 5c). Competition ELISA performed using integrin and Ab17 indicated a reduction in Ab17 binding to VLPs pre-coated with integrin compared to VLPs not pre-coated with integrin (Supplementary Fig. 7). The inverse, a reduction in integrin binding after pre-coating of VLPs with Ab17, was also true (Supplementary Fig. 7) showing that integrin and full antibody compete for binding on the VLP surface.

To develop a mechanistic understanding of antibody function, the binding and neutralisation of the tri-serotype specific antibodies as Fabs and full antibodies was compared. For all three serotypes, Fabs exhibited weaker avidity with similar or faster association and much faster dissociation rates as expected (Supplementary Fig. 2 and Supplementary Tables S8 vs S3). However, despite similar BLI responses, Fabs were not capable of neutralising FMDV even at the highest concentration tested (2.5 µM), a value well beyond biological significance. This indicates that the full antibody is required to neutralise viral activity. To determine whether direct binding to the G-H loop is part of the neutralisation mechanism, the tri-serotype specific antibodies were tested for binding to both standard A22 VLPs and A22 VLPs lacking the VP1 G-H loop (also termed ‘loopless’, LL). The binding kinetics for both were similar and, although the BLI nm response was significantly decreased for the A22 LL VLPs (Supplementary Fig. 8), the antibodies were still capable of binding to them. This suggests that the G-H loop does not form part of the antibodies core epitope. Additionally, most F(ab´)_2_ fragments (IgGs minus Fcs) failed to neutralise the virus at concentrations up to 2.5 µM or at an over 200-fold molar excess as compared to the amount required of full antibody (Supplementary Table 10), indicating that bivalent binding is unlikely to contribute to the mechanism of neutralisation of the tri-serotype specific antibodies. Steric RGD blocking by the volume of the full antibody, abolishing integrin-dependent cell entry, is therefore a plausible neutralisation mechanism.

## Discussion

We sequentially vaccinated cattle with FMDV antigens from four serotypes, and then characterised the vaccine-induced antibodies, demonstrating a cross-specific response. Overall, the ELISpot data mirror the response observed by Grant and colleagues in a similar FMD sequential vaccination study^45^ with the notable difference that we observed a cross-reactive response to Asia1S and to a lesser extent A22 and SAT2Z from an O1M homologous boost. This could be attributed to the use of intact VLPs to detect antigen-specific cells in our assay, increasing the assay’s sensitivity compared to previous studies. From these results and those of Grant et al.^45^, we noticed a lower antigen specific B cell response after the prime vaccination as opposed to the booster. However, the prime vaccination led to the correct detection of antibodies by VNT and LPBE. Certainly, a large amount of antibody can be secreted from even a few ASCs even those below the detection limit by ELISpot. These cells may also be missed on sampling whereas antibodies are more likely to be diffuse throughout the animal’s serum. LPBE and VNT failed to detect a cross-reactive response after O1M boost, suggesting that the cross-reactive B cells present after the O1M homologous boost produce low levels of antibodies, which fall below the detection levels of these assays. After the first heterologous boost, the cross-reactive response by VNT and LPBE is more evident particularly for Asia1S; however only low numbers of Asia1S ASCs are observed. This is likely due to small numbers of cross-reactive B cells producing high levels of antibody in the serum and highlights the utility of examining all three assays in parallel to assess cross-reactivity post vaccination.

By a series of triage steps, 350 antigen-specific B cells with varying specificities identified based on their ability to bind FMDV VLPs were narrowed down to 24 FMDV-specific antibody secreting cells; of these, 15 produced antibodies which were specific for a single serotype. Ten of the single serotype-binders were O1M-specific and two of these were unable to neutralise O1M virus. This raises the question as to the functions of these non-neutralising strong binders in response to FMD vaccination. Opsonisation titre for example has been shown to be as predictive of protection as neutralisation titre^67^, and highlights that many questions remain regarding the complex interplay between neutralising and non-neutralising antibodies in the protective response against FMDV. While neutralising antibodies are crucial in directly blocking virus infection, non-neutralising antibodies may still contribute to the overall immune response, although the exact requirements to mount a protective response against FMDV remain unclear. Six of the 24 FMDV-specific antibodies were bi-specific, binding either O1M and Asia1S or A22 and Asia1S, with none bispecific for O1M and A22 (in contrast with the O/A bi-serotype specific antibody identified by He et al.^35^); this is probably due to limited sampling. Finally, three antibodies, Ab17, Ab34, and Ab49, were specific for three serotypes, O1M, A22, and Asia1S. These showed high avidity, in the following order: O1M > A22>Asia1S, likely mirroring the vaccination regime order^68^. This hierarchy may therefore be a consequence of the additional dose of O1M or immunological imprinting from the primary immunisation and other immunisation strategies would likely result in different antibodies in their totality. The order chosen for immunisation was based on a previous study successfully eliciting cross reactivity^45^. They exhibited broad potency, being able to neutralise 24 virus topotypes from serotypes O, A, Asia1, and C. These antibodies also bound an additional 41 viruses of the same serotypes when screened by ELISA, further validating their broad binding capabilities. The hierarchy of binding (O > A>Asia) observed using VLP assay was broadly maintained when using live virus. We anticipate that these 41 viruses would also be neutralised by Ab17, Ab34, and Ab49.

Competition BLI assays revealed that the three tri-serotype specific antibodies compete for binding to the same antigenic region and cryo-EM analysis of their Fabs in complex with VLPs showed that all three bind the C-terminal peptide of VP1 essentially indistinguishably, dislodging some of the C-terminal region from the VLP surface to bind in a flexible way. The overlap of the Fab density with the volume occupied by bound integrin receptor^56^, coupled with competition ELISA data showing a reduction in binding of either antibody or soluble integrin when VLPs are pre-incubated with the competitor, suggests that the antibodies would inhibit receptor binding, particularly considering the less flexible arrangement when integrin is fixed on the cell surface. The preservation of binding of Ab17, Ab34, and Ab49 in the absence of the G-H loop, confirms that inhibition is not due to direct binding of antibody to the G-H loop. Furthermore, BLI and VNT analyses using the Fab format of Ab17, Ab34, and Ab49, revealed that despite having similar binding responses to the full antibodies, Fabs were unable to neutralise the virus even at the highest concentration tested (2.6–2.65 µM), indicating that steric hindrance of integrin receptor binding by the full antibody is the likely mechanism of virus neutralisation. This interpretation is further supported by the observation that most F(ab´)₂ fragments, which retain bivalent binding but lack the Fc region, also failed to neutralise the virus at equivalent concentrations or at an over 200-fold molar excess compared to the concentration required of the full antibody. These findings suggest that neither monovalent nor bivalent antigen engagement alone is sufficient for neutralisation. The steric volume of the intact antibody is likely critical for blocking integrin binding and therefore viral entry. Structural modelling also suggests that the two Fab arms could reach between adjacent VP1 C-termini without occluding the integrin binding G-H loop, further highlighting steric interference as the primary neutralisation strategy.

SPR studies with peptides confirmed the C-terminal region of VP1 as the likely site of antibody attachment with motif ^203^QxI/LxAPxK^210^ conserved in all bound viruses. This led us to further investigate the structural basis of binding, first by crystallographic analysis of all three Fabs in isolation, which revealed considerable structural variation, notably in the long CDR-H3s (a common feature of cattle antibodies), but also a common groove with conserved charge features. Co-crystallisation of the Fab of the most potent antibody (Ab34), with VP1 O1M C-terminus-derived peptides revealed their binding into this groove. Interestingly, the peptides were largely hydrophobic and fully extended, a conformation that allows them to form a short β-strand which clips onto part of the CDR-H3, in a side-chain independent fashion. The core linear epitope, located within the C-terminal VP1 peptide, can only engage with the antibody after being pulled away from the virus surface. This exposes the hydrophobic surface of the peptide which is then available to be buried into the groove on the antibody. This peeling away of the C-terminal amino acids from the capsid surface may explain the observation that some non-binder viruses that contain the same or very similar core binding motif are not recognised by these antibodies (e.g., C/NEP and C/KEN, whilst SAT1, SAT2, and SAT3 viruses do not possess the ^203^QxI/LxAPxK^210^ peptide motif). It is likely that underlying residues will modulate the strength of attachment of the C terminal epitope to the capsid surface, with a more strongly attached epitope being less available for antibody binding. This is supported by the observation that some peptides derived from non-binder viruses bind these antibodies in SPR.

Comparing the grooves on the three antibodies, it seemed that all might bind to the same region of the VP1 C-terminus and in a common fashion, and we used Alphafold3 to further test this^59^. Encouragingly, the software almost exactly reproduced the binding of the core 7-residue peptide to Fab34 and then predicted the same binding pose to the other two Fabs from Ab17 and Ab49, despite significant variation in the structures of these antibodies, most notably in the heavy chain CDR3 loops, which differ in length and have three distinct patterns of stabilising disulphide bonds.

In summary, the highly unusual breadth of reactivity of the tri-serotype specific antibodies is generated by reusing a cryptic conserved hydrophobic surface, and focussing significantly on side chain independent interactions, although the side chains of VP1 residues Q203, I205, A207, and K210 are also important for the interaction. We note that there are now examples of unusual broadly cross-reactive antibodies against several viruses and the re-use of cryptic hydrophobic surfaces is a common theme^33,69,70^. The VP1 C terminus had previously been identified as an immunodominant FMDV epitope which induced cross-reactivity, however the neutralisation ability of antibodies targeting this area was previously unclear^38,41,44^. The combination of the previously identified peptide (200–213)^38^ and our refined epitope mapping (203–210) suggests the existence of a single cross-reactive epitope recognised across FMDV serotypes O, A, and Asia1. While it has often been very difficult to generate such cross-reactive antibodies as described here, the inherent flexibility of the C-terminus of VP1 in many FMDVs probably facilitates presentation of the epitope. However, we observed that certain viruses, despite bearing a C-terminal sequence that is recognised by the antibodies when presented as a peptide, do not bind the antibodies in the context of the complete virion, perhaps due to stronger interactions with the capsid’s surface arresting the presentation of the epitope.

## Methods

### FMDV antigen and viruses

Serotypes used in the animal experiment were described in Clarke et al.^33^. Briefly, antigens used to vaccinate calves were double purified, inactivated, FMDV O_1_/Manisa/TUR/69 (O1M), A_22_/IRQ/24/64 (A22), Asia1/Shamir/ISR/89 (Asia1S) and unpurified baculovirus-produced SAT2/SAU/6/2000 (SAT2S) VLPs (virus like particles). Additionally, highly purified baculovirus-produced SAT2/ETH/65/2009 (SAT2E) VLPs were used to test binding of antibodies by BLI.

Thermo- and pH-stable FMDV VLPs, carrying a Cysteine at position 93 in VP2 were produced using either a vaccinia virus production system (VAC) or a baculovirus expression vector system (BEVS), as previously described^49,50^. The VAC system was used to express strains O1M, A22, Asia1S and SAT2/ZIM/7/83 (SAT2Z) since no VAC compatible SAT2S construct was available. O, A, and Asia1S VAC derived VLPs used in this study bear a VP2 93 C stabilising mutation at the 2-fold axis, SAT2Z bears a 93Y mutation. Briefly, HEK293 cells were co-infected with a vaccinia virus encoding an FMD virus-derived P1-3C cassette (Multiplicity of infection, MOI 10) and a T7 polymerase-coding vaccinia virus recombinant vTF7.3 (MOI 5). After 24 h, infected cells were harvested, lysed, and the lysate pelleted through a 30% sucrose cushion. The extract was loaded onto a 15–45% sucrose gradient and spun for 17 h at 23,000 rpm in rotor SW32 Ti (Optima L100-XP, Beckman Coulter) at 10 °C. The 36 mL gradient was fractionated into 1 mL aliquots and analysed using SDS-PAGE on 4–12% acrylamide Novex NuPAGE gels (Invitrogen, Thermo Fisher Scientific). Fractions where bands corresponding to VLPs were observed were pooled, and the concentration was determined with a Qubit 2.0 Fluorometer (Thermo Fisher Scientific). When required, samples were concentrated by ultrafiltration with 100 kDa cutoff Amicon centrifugal filter units (Merck) following the manufacturer’s instructions. VLPs were used as the capture antigens in ELISpot, bio-layer interferometry (BLI), and ELISA assays.

For BLI with SAT2E and cryo-EM complexing with O1M and A22, VLPs also carrying the VP2 93 C mutation were produced via the BEVS as described previously^48^. P1-3C expression cassettes under the control of promoter p10 were recombined into bacmid Flashbac Gold (Oxford Expression Technologies) and the resulting baculoviruses were used to infect Tnao38 insect cells at a MOI of 1. Harvest was three days post -infection and the VLP purification process was essentially as described above for the VAC expression system. In the case of SAT2S, also produced *via* the BEVS, the 93 C VLPs were too unstable for successful purification hence usage of a crude supernatant for cattle immunisation.

Polyclonal sera and viruses used in VNTs were obtained from the FAO World Reference Laboratory for FMD (WRLFMD, The Pirbright Institute).

SAT2 SAU/6/2000 (AF367135), ZIM/7/83 (DQ009726) and ETH/65/2009 (KF112945) VP1 amino acid sequences were aligned using BioEdit (v 7.2.5^71^) and are shown in Supplementary Table 1.

### Sequential immunisation of cattle with FMDV antigens

The animal experiment has been described previously^33^. Briefly, four male 5-6 month-old Holstein-Friesian calves (*Bos taurus*) obtained from the Centre for Dairy Research (CEDAR, University of Reading) were vaccinated with 10 μg of double purified inactivated vaccine strain O1M for the prime vaccination at day 0 and then sequentially received 6–8 µg of O1M, 10 μg of A22, 10 μg of Asia1S double purified vaccine strain antigens, and 10 µg of unpurified SAT2S VLPs on days 21, 42, 63, and 84 respectively after primary vaccination (Supplementary Table 11). All immunisations were formulated in Montanide ISA 206 adjuvant (SEPPIC) with 50 mM HEPES and 100 mM KCl and administered intramuscularly above the right prescapular lymph node. Two control calves were administered an adjuvant-only immunisation at each timepoint.

Heparinized blood and serum samples were obtained from each calf at every time point following the primary and boost vaccinations. Calves were culled 105 days after the primary vaccination; right and left prescapular lymph nodes were collected from all calves, and the sternum bone marrow was collected from vaccinated calf three. All experiments were approved by The Pirbright Institute’s ethical review process and followed national guidelines on animal use.

All animal blood sampling and euthanasia procedures were reviewed and approved by the Animal Welfare Ethical Review Body at The Pirbright Institute, and the internal ethics committees at the Centre for Dairy Research (CEDAR, University of Reading), or the Animal and Plant Health Agency (APHA). Experiments were conducted in accordance with the UK Animals (Scientific Procedures) Act 1986 under project licence PPL 7998958 and complied with the ARRIVE (Animal Research: Reporting of In Vivo Experiments) guidelines. Briefly, cattle were sedated with an intramuscular injection of 4 mL Xylazine Hydrochloride (Rompun®). Once laid down, typically within five minutes, euthanasia was carried out by intravenous administration of 100 mL Pentobarbital sodium (Euthatal®). Confirmation of death was based on the absence of a detectable pulse or respiratory movements, no corneal reflex, and lack of an audible heartbeat using a stethoscope. If any vital signs remained, additional 50 mL of Euthatal® were administered until death was confirmed.

### Cattle peripheral mononuclear cells (PBMC), plasma and sera preparation

Cattle PBMCs were isolated from heparinized blood samples as described in Clarke et al.^33^. A fraction of cells was resuspended in 10% Horse Serum DMSO (Sigma-Aldrich) for cryopreservation and another fraction was diluted in ELISpot medium and used in the preparation of ELISpot plates. Plasma was recovered from the heparinized blood samples after gradient separation.

For serum collection, blood samples were left to stand at room temperature for at least 30 min before being spun at 2000 x *g* for 30 min. The sera were recovered and stored at −80 °C until further use.

### Liquid Phase Blocking ELISA (LPBE)

Liquid phase blocking ELISA (LPBE) was used to determine the titres of circulating antibodies against FMDV in the plasma of the animals after vaccination. This was carried out by the WRLFMD as previously described^47,72^.

### Virus neutralisation test (VNT)

A virus neutralisation test (VNT) was performed by the WRLFMD to identify the presence of anti-FMDV neutralising antibodies in the plasma samples. Briefly, serial dilutions starting from 1/4 (final starting dilution becomes 1/8 after addition of the virus) of sera were incubated for 1 h at room temperature with 100 TCID50 of infective FMDV O1M, A22, Asia1S, and SAT2S. The virus plasma mixture was overlaid with an IB-RS^2^ cell suspension at a concentration of 1 × 10^6^ cells/mL. The plates were then sealed and incubated at 37 °C. Cytopathic effects were observed after 24–72 h. Titres of virus-neutralising antibodies were expressed as log_10_ of the reciprocal of the sera dilution, which neutralises 50% of the wells.

Similarly, the 50 purified recombinant antibodies were titrated for viral neutralising activity against the three FMDV serotypes following the procedure described above, except where Ab17, Ab34 and Ab49 were serially diluted from neat concentration with viruses A/IRN/87- and A/IRN/87+.

A panel of 37 most recently circulating topotypes belonging to the seven antigenically and genetically divergent serotypes of FMDV: 11 O, seven A, four C, four Asia 1, and 11 from SAT1, SAT2, and SAT3 were selected and used in a VNT assay to determine the neutralisation potential of the tri-serotype specific antibodies.

### FMDV-specific ELISpot assay for the detection of antibody secreting cells (ASC)

An FMDV-specific ELISpot assay using freshly isolated PBMCs was adapted from Grant et al.^45^. Briefly, MultiScreen HA plates (Millipore, Merck) were coated for 2 h at 37 °C with 2.5 μg/well of purified FMDV VLPs (O1M, A22, Asia1S, and SAT2Z), 10 µg/mL Conalbumin (Con A), or a 1/1000 dilution of anti-total cattle IgG (clone BG-18; B6901 Sigma-Aldrich) diluted in 0.1 M carbonate buffer (pH 9.6). Plates were blocked overnight using 4% milk in PBS-Tween (0.05% tween) and after a washing step, 100 μl of PBMCs were seeded at 5 × 10^5^, 1.25 × 10^5^ and 3.125 × 10^4^ cells/well in duplicate in culture medium made of RPMI containing Glutamax and 25 mM HEPES (Gibco), supplemented with Penicillin-Streptomycin (100X, Gibco); 1 mM sodium pyruvate (Gibco) and 1X non-essential amino acids (Gibco) and incubated over night at 37 °C. FMDV-specific ELISpots were detected by incubation with anti-cattle IgG conjugated to horseradish peroxidase (HRP) (Bio-Rad). The assay was developed using 3-amino-9-ethylcarbonate substrate (AEC) (Merck) and analysed using the AID ELISpot reader (Autoimmun Diagnostika GmbH), which allows for the automated counting of spots based on size and intensity. Each spot equates to an ASC. The ELISpot assay results were manually validated for false positives and expressed as the number of ASCs per 10^6^ PBMCs (mean of duplicate wells). During the ELISpot data analysis, wells with less than two spots were considered negative (minimum sensitivity, four ASCs per 10^6^ PBMCs).

### Single cell antibody discovery workflow

PBMCs were thawed and activated in culture to generate memory B cells prior to injection into AbCellera’s microfluidic screening devices with nanoliter-volume reaction chambers. FMDV-specific ASCs were identified using a multiplexed bead-based assay, consisting of VAC derived O1M, A22, Asia1S and SAT2Z VLPs-conjugated optically to beads. Porcine parvovirus VLP-conjugated beads were used as a counter screen. Binding of secreted IgGs to antigen-conjugated beads was detected via a fluorescently labelled anti-bovine IgG secondary antibody, and positive hits were identified with machine vision and recovered using automated robotics-based protocols. Next-generation sequencing libraries (MiSeq, Illumina) were produced using single-cell polymerase chain reaction (PCR) and custom protocols with automated workstations (Bravo, Agilent). Paired heavy and light chain sequences for each recovered ASC were obtained using a custom bioinformatics pipeline. Each sequence was annotated with the closest germline (V(D)J) genes, degree of somatic hypermutation, and potential sequence liabilities. Antibodies with the same CDR-H3 length and inferred heavy and light V and J genes were considered members of the same clonal family.

### Expression and purification of selected cattle antibodies using HEK293 cells

The selected antibodies were recombinantly generated as cattle IgG1/LCλ isotypes as described previously^73^. Briefly, VDJ heavy and VJ light coding fragments of FMDV specific antibodies were ordered as synthetic gene blocks (IDT) and directionally cloned into corresponding pNeoSec-BovFc-IgG1 and pNeoSec-BovLC-λ expression vectors using in-fusion ClonExpress II One Step Cloning Kit (Vazyme) as per manufacturer’s instructions. The IgG1 and IgL vector pairs were then transiently co-expressed in Expi293F^TM^ cells (Gibco) at 5 mL scale in 50 mL mini bioreactors (Corning) according to Expi293 transfection protocol^74^ (without addition of kifunensine to tailor glycosylation) and the Gibco Expi293 Expression System User Guide (Thermo Fisher Publication Number: MAN0007814). Briefly, 2.5 μg of the plasmid DNA constructs for both the heavy and 2.5 μg of the corresponding light chain were mixed in 0.5 mL Opti-MEM (Gibco, ThermoFisher Scientific) containing 27 μg of polyethylenimine (PEI) 40 K MAX (Polysciences) and added to 1 × 10^7^ of Expi293™ cells seeded in 5 mL culture volume. After 18 h, valproic acid, sodium propionate, and glucose enhancers were added to the transfected cultures which were then incubated for 3 days before harvesting the antibodies from the culture supernatant^73,75,76^. The presence of bovine IgG was determined by western blot analysis following SDS-PAGE (4–12% Bis-Tris gel, NuPAGE) under non-reducing conditions using 1/10,000 diluted sheep anti-bovine IgG HRP (BioRad) with ECL Prime Reagent substrate (Cytiva). Selected antibodies were scaled up for expression at a 300 mL level followed by purification on an AKTA pure 25 using 5 mL Protein G chromatography (Cytiva), and then buffer exchanged into Dulbecco’s PBS (DPBS) without calcium and magnesium. The concentration in mg/mL of the purified antibodies was determined by A280/1.4 and the antibodies were diluted to 1 mg/mL in DPBS.

### Production of recombinant Fab and F(ab´)_2_ fragments

Antibodies 17, Ab34, Ab49, Ab122 were recombinantly expressed as Fab and F(ab´)_2_ fragments fused to a six Histidine (6xHis) tag at the C-terminus^77,78^. The gblocks for the heavy and light chains variable regions were correspondingly cloned into pOPINBOVH and pOPINBOVL for cattle Fab expression^76^ and into a newly build pNeoSec-BovFab2 and a pNeoSec-BovLC-λ for expression as cattle F(ab´)_2_. The pNeoSec-BovFab2 vector was designed based on the cattle IgG1 constant region sequence (accession number S82409.1) used in the pNeoSec-BovFc-IgG1 vector, carrying CH1 constant domain, a hinge region and first 8 amino acids of CH2 (predicted using ScanProsite and IMGT annotation tools) to allow dimerisation of Fab fragments by disulphide bridging between three cystines present in cattle IgG1 hinge region. The cloning and co-expression of corresponding heavy and light chain pairs were performed as described for full antibodies. The expression of Bovine Fab and F(ab´)_2_ was determined by Western blot analysis under non-reducing conditions using mouse anti-His tag antibody (Dilution: 1/500; BioRad) probed by goat anti-mouse IgG HRP (Dilution: 1/10,000; Invitrogen) for Fabs detection whereas F(ab´)_2_ fragments were screened using sheep anti-Bovine IgG antibody (Dilution: 1/10,000; BioRad) probed by donkey anti-sheep IgG HRP (Dilution: 1/20,000 Invitrogen) in the presence of substrate ECL Prime Reagent (Cytiva). Following expression confirmation, the selected fragments were scaled up for expression at 300 mL in Expi293™ cells. The Fab fragments were purified on the AKTA pure 25 chromatography system using a 5 mL HisTrap column (Cytiva), and then buffer exchanged by dialysis into DPBS without calcium and magnesium. Concentration of the purified Fab in mg/mL was determined by A280/1.3-1.4 and raised to ≥15 mg/mL using Amicon Ultra 10 kDa molecular weight cut-off concentrators (Merck, Gillingham, UK). The F(ab´)_2_ proteins were purified in two steps, the supernatants were first processed on the AKTA pure 25 chromatography system using a 5 mL Protein G HP column (Cytiva) to bind both F(ab’)_2_ and monomers, followed by HiLoad 16/600 Superdex 200 pg size exclusion chromatography (Cytiva) using DPBS without calcium and magnesium as the running buffer to separate Fab dimers from monomers. Concentration of the purified F(ab´)_2_ in mg/mL was determined by A280/1.3–1.4 and raised to 1 mg/mL using Amicon Ultra 10 kDa molecular weight cut-off concentrators (Merck).

### Bio-layer interferometry (BLI) binding assay

BLI was used to measure the VLP-antibody complex formation at the sensor surface (response in nm) as well as the VLP-antibody association, dissociation, and equilibration dissociation constants (Ka, Kdis, KD) to assess the serotype-specificity of each of the 50 expressed antibodies. The BLI assays were performed on an Octet RED384 instrument (Sartorius AG, Sartorius AG) at 30 °C with shaking at 1000 rpm, using streptavidin (SA) coated biosensors (Sartorius AG). The purified FMDV antigen (stabilised O1M, A22, Asia1S and SAT2Z VLPs or stabilised A22 G-H-loopless VLPs) was biotinylated following the Lightning-Link® Fast Type B protocol (ab201796, Abcam) using a 2:1 ratio of VLP:biotin. Before use, SA biosensors were loaded into the columns of a biosensor holding plate and pre-hydrated in HBS-EP buffer (BioQuote) for 20 min. Optimal concentrations of biotinylated-VLP, referred to as the ligand, were determined by scouting experiments. The ligand was diluted in HBS-EP buffer and each dilution (ranging from 175 pM to 1.75 nM) was evaluated for its loading ability and production of a binding signal (>0.1 nm) when an antibody or FAb solution (analyte) was added.

The final assay included the following steps: baseline/equilibration for 60 s (HBS-EP buffer); loading for 450 s (438 nM biotinylated VLP in HBS-EP buffer); washing for 60 s (HPS-EP buffer); second baseline for 60 s (HPS-EP buffer); association for 1200 s (with antibody/Fab solution at 25 nM, 50 nM, and 100 nM); and dissociation for 1200 s (HPS-EP buffer). The experimental data were fitted with the 1:1 binding model and analysed with global fitting using Octet Data Analysis 9.0 software to determine antibody avidity. A reference sample (VLP + no antibody) and reference sensor (no VLP + antibody 100 nM) were included in each assay as controls for non-specific binding. Polyclonal sera (supplied by WRLFMD) from cattle vaccinated with the corresponding serotypes were used as positive controls, and a cattle antibody specific to bovine respiratory syncytial virus, bRSV (B4 antibody,^76^) was used as negative control. A cut-off response value of 0.1 nm was calculated using RCutoff in RStudio (v4.4.0) and used to discriminate binder from non-binder antibodies.

For the binding-competition assay, the streptavidin-biotinylated VLP coated sensors were incubated with the first antibody at saturating concentrations (400 nM). Then the biosensors were immersed into the second competing antibody solution (400 nM) for the same incubation time. Raw data were processed using ForteBio’s Data Analysis Software 7.0. Additional binding by the competing antibody indicates an unoccupied epitope (non-competitor), while no binding indicates epitope blocking (competitor).

### FMD virus ELISA

To investigate the broad binding capability of Ab17, Ab34, and Ab49, 76 FMDV isolates were selected that were representative of FMDV antigenic and genetic diversity. Integrin homologous sandwich ELISAs were performed to account for virus antigen content. All plates were coated with bovine αvβ6 integrin (1 µg/ml) in PBS (phosphate buffered saline containing cations, 2 mM CaCl_2_ and 1 mM MgCl_2_) at 50 μl/well and incubated overnight at 4 °C. Integrin sandwich ELISA plates were then washed three times with PBS-Tween (0.05% tween) and blocked with 2% casein in PBS-Tween for 1 h at room temperature (rt), 100 μl/well. Wells were washed as before and 50 μl virus S/N diluted 1:1 in PBS (plus cations) were incubated for 1 h at rt. After washing, 50 μl of 0.5 μg/mL integrin-HRP in PBS-Tween (0.05% tween) was added for 1 h at rt. For bovine mAb ELISA, plates were blocked with 20% horse serum in PBS-Tween for 1 h, 100 μl/well. Wells were washed as before and 50 μl virus S/N diluted 1:1 in PBS (plus cations) were incubated for 1 hr After washing, 50 µl of 1 µg/mL bovine mAb in PBS-Tween was added for 1 h, then wells were washed again and incubated with anti-bovine HRP secondary antibody (at 1/10,000) in PBS-Tween for 1 h. All plates were again washed and 50 μl TMB substrate added for 10 min at rt. The reaction was stopped with 50 μl acid (0.6 N sulphuric acid) and plates were read at 450 nm. Background binding of negative control antibody B4 was subtracted from Ab17, Ab34, and Ab49 absorbance values. Antibody to integrin binding ratios were calculated by dividing antibody absorbance by integrin absorbance. Cutoff values for non-binders were calculated using the RCutoff package in R studio (v4.4.0) as an absorbance of 0.17 nm.

### Integrin competition ELISA

Competition ELISA involving αvβ6 integrin and recombinant antibodies was performed to determine whether tri-serotype specific antibodies were blocking the integrin binding site of the FMDV VLP. Nunc Immunosorb ELISA plates were coated with stabilised O1M VLP in sodium bicarbonate coating buffer at 4 ^o^C overnight. Antibody or integrin was then applied in molar excess and incubated for 1 h at room temperature with shaking at 120 rpm. After washing, biotinylated competing molecule (integrin or antibody) was added and incubated for the same time. Detection was achieved using streptavidin-HRP (BioRad) diluted 1/10,000 and incubated for 1 h at room temperature with shaking. ELISAs were developed using TMB and stopped using ELISAStop (Invitrogen). Antibodies, integrin, and streptavidin-HRP were all diluted in 1% milk (Marvel) in PBS-Tween (0.05 % tween 20). Three washes in PBS-Tween were performed between each step. Bovine serum known to bind O1M VLP was used as a positive binding control, with anti-bovine RSV antibody B4 used as a negative binder. Antibody pairs known to block or be permissive were also included in the assay.

### Western blotting

Western blotting was performed as described previously^33^. Briefly, reduced O1M, A22, and Asia1S VLPs produced *via* vaccinia virus were run on 4–12% Bis-Tris SDS-PAGE gels, then transferred to using the iBlot2 dry blotting system using a nitrocellulose membrane (Invitrogen). Primary antibody (Ab17, Ab34, Ab49, Ab117 and B4) was diluted to 0.2 µg/mL in 1% milk PBST and incubated on the blot for 1 h at room temperature. Secondary sheep anti-bovine-HRP (BioRad) was used at a 1/10,000 dilution in PBS-Tween with 1% milk and incubated on the blot for 1 h at room temperature. For mouse-anti-VP2 antibody 4A3, a dilution of 1/5000 was used, and goat anti-mouse-HRP (BioRad) diluted 1/10,000 was the secondary antibody. After three washes, blots were developed using ECL Prime (Amersham) according to the manufacturer’s instructions and imaged on the ChemiDoc system (BioRad).

### Antibody peptide interaction ELISA

Peptides 277, 278, and 279, representing residues 191 to 213 of O/UKG/34/2001, were ordered lyophilised from Thermofisher Scientific and reconstituted in 100% DMSO to a concentration of 5 mg/ml. Nunc Maxisorp ELISA plates were coated with 0.5 µg/mL of peptide in PBS and adsorbed for 1 h at 37 ^o^C. This was followed by biotinylated primary antibody (Ab17, Ab34, Ab49, Ab117) at 5 µg/mL in PBS-Tween (0.05% tween) + 1% milk, and then streptavidin-HRP (Bio-Rad) at a dilution of 1/10,000, both for 1 h at rt. Plates were washed three times with PBS-Tween between each step. To develop 100 µL of TMB was added, with an equivalent volume of ELISAStop (Thermofisher Scientific) used to quench the reaction. Absorbance at 450 nm was read with absorbance at 630 nm subtracted.

### Surface Plasmon Resonance (SPR) peptide-Ab interaction

To investigate peptide antibody interactions peptides were made to order by BioServ UK (5-mer and 12-mer) and reconstituted in PBS. SPR assays were performed using the Carterra LSA-XT system. A HC30M chip (Carterra) was functionalized with successive cycles of 50 mM NaOH, 1 M NaCl, and 10 mM glycine pH 2.0. Carboxyl groups on the chip surface were activated using a 1:1:1 ratio of 400 mM EDC/100 mM NHS/PBS (Cytiva). Antibodies were diluted in 10 mM NaOAc (Carterra) and coupled to three independent locations on the chip over a 10 min duration. Free active carboxylate groups were quenched using 0.5 M ethanolamine (Carterra). Peptides were diluted to 10 µg/mL in running buffer (PBS + 0.005% tween) and injected sequentially allowing 5 min association and 15 min dissociation. The antibody coated chip was regenerated between each peptide using three 30 s injections of glycine pH 2.0. Data was analysed using Carterra kinetics software, maximum response units (RUMax) was calculated as the average response (RU) immediately before the dissociation phase. To validate results, the assay was also repeated using two higher capacity HC200M chips. The maximum binding response (RUMax) was calculated immediately before the dissociation phase of the assay and averaged across the three chips.

### Sequence logo generation

Sequence logos of the tri-serotype specific binding motif were generated using the ggseqlogo package^79^ in R studio (http://www.rstudio.org/).

### Crystallisation, X-ray data collection and structure determination

Fabs were expressed independently of the full antibodies and purified. Fabs were crystallised using a series of screens (Hampton) in Crystalquick 96-well X plates (Greiner Bio-One). Distribution into wells was with a Cartesian Robot using the sitting-drop vapour-diffusion method, with 100 nL protein and 100 nL reservoir solution in each drop^80^. The best diffracting crystals were found to be in well B9 of the PEGRx screen (0.1 M bicine pH 8.5, 15% Polyethylene glycol (PEG) 1500) for Fab17, Hampton Wizard screen 1 reagent 41 (0.1 M CHES pH 9.5 30% w/v PEG 3 K) for Fab34 and PEGRx well D2 (9.1 M imidazole pH 7.0, 20% w/v PEG 6000) for Fab 49.

To obtain Fab/peptide complexes, peptides were made to order by BioServ UK (7-mer) and GenScript (18-mer), each delivered lyophilised. Both peptides were dissolved in minimal 10 mM Tris pH 7.5 buffer, before complexing with Fab34 in a 10-fold molar excess of peptide to Fab. Trays were set up shortly after complex solutions were prepared using sitting drop vapour diffusion as above and left at room temperature. For the 7-mer structure, crystals were observed in the first well of the JCSG+ crystallisation screen (0.2 M Lithium sulfate monohydrate, 0.1 M Sodium acetate trihydrate pH 4.5, 50% v/v PEG 400). For the 18-mer structure, diffracting crystals were found in PEGRx well C8 (0.1 M MES monohydrate pH 6.0, 14% w/v PEG 4000).

Crystals were mounted in loops and dipped in solution containing 25% glycerol and 75% mother liquor for about one second before freezing in liquid nitrogen. Diffraction data were collected at beamline I03 of Diamond Light Source, UK, using the queue system that allows unattended automated data collection (https://www.diamond.ac.uk/Instruments/Mx/I03/I03-Manual/Unattended-Data-Collections.html). We collected 3600 diffraction images of 0.1° each at 100 K from a single crystal for each data set. Data integration, scaling and reduction were automatically done with Xia2-dials^81–83^. The structures were determined using molecular replacement with Phaser^84^; model rebuilding was done with COOT^85^ and refinement with Phenix^86^. There are two Fabs in the crystallographic asymmetric unit of Fab34. Density for residues 102-106 of CDR-H3 in one Fab and residues 53-54 of CDR-H2 in the other Fab is poorly defined, models for these residues were not built. There are six complexes in the crystallographic asymmetric unit of Fab34/18-mer peptide. Density for the constant domains of two of these Fabs is weak due to flexibility and lack of crystal contact. Data collection and structure refinement statistics are given in Supplementary Table 8. Structural comparisons used SHP^87^ and figures were prepared with PyMOL (The PyMOL Molecular Graphics System, Version 1.2r3pre, Schrödinger, LLC).

### Cryo-EM sample preparation, data collection and image processing

VLPs used for the generation of complexes had been produced in insect cells using the BEVS and were O1M FMDV with a stabilising mutation at the 2-fold axis in the VP2 unit residue 93 (cysteine). Fab/FMDV complexes at a molar excess of 300/1 were made using 0.3 mg/mL of FMDV VLPs and incubated for 30 s on ice. Prior to vitrification, 2/1 copper 200 mesh Quantifoil grids with a 2 nm carbon layer were glow discharged for 30 s on ‘high’ (Harrick Plasma). Complex material was then applied within a chamber at 4 °C and at 100% relative humidity, blotted (6 s with a + 6 force, 30 s wait) and vitrified with liquid ethane using a vitrobot mark IV (Thermofisher Scientific). Movies were acquired using EPU software on a Krios G3i microscope operating at 300 kV, equipped with a Falcon-4i SelectrisX detector/energy filter (Thermofisher Scientific) at 130 kX magnification, corresponding to a calibrated pixel size of 0.932 Å/pix and written in EER format. A total dose of 49.9 e^-^/Å^2^ was applied, EER files were split into 50 frames and a defocus range of −0.8 to −2.6 (0.3 μm increments) was used.

Data were pre-processed on the fly using cryoSPARC live^33^. A total of 7529 movies were collected for Fab34, 10,000 for Fab49 and 11,486 for Fab17 all in complex with O1M VLPs bearing a VP2 93 C thermostabilising mutation at the 2-fold axis. For Fab17, particles were blob-picked from the initial 1000 movies of the Fab17 complex, and the resulting 2D class averages were then used for template-picking for all three fabs. This resulted in initial particle sets of 27,242, 63,153 and 36,434 particles for Fab17, 49 and 34, respectively. Decoration around the VLP was visible in processed micrographs and subsequent 2D class averages, however in the 3D reconstructions the signal for Fab was weak (Supplementary Fig. 5). Models of O1M (PDB 5NE4) were first rigid body fitted into each map using Coot^77^ before refinement of a ‘7-mer’ i.e., seven icosahedral asymmetric units such that a single unit was surrounded by contacting subunits to account for clashes but avoid computationally costly full VLP refinement in the Phenix real space refine module, Supplementary Table 8^84^.

### Statistics and reproducibility

Throughout these analyses, the Wilcoxon matched-pairs signed rank test was used as appropriate and two-tailed P values were calculated (see figure legends). No statistical method was used to predetermine sample size. No data was excluded from the analysis. The experiments were not randomised. The Investigators were not blinded to allocation during experiments and outcome assessment.

### Ethical approval

All animal blood sampling procedures were reviewed and approved by the relevant ethics committees at The Pirbright Institute, the Centre for Dairy Research (CEDAR, University of Reading), or the Animal and Plant Health Agency (APHA). Experiments were conducted in accordance with the UK Animals (Scientific Procedures) Act 1986 under project licence PPL 7998958 and complied with the ARRIVE (Animal Research: Reporting of In Vivo Experiments) guidelines.

### Reporting summary

Further information on research design is available in the Nature Portfolio Reporting Summary linked to this article.

## Supplementary information

Supplementary Information

## Data Availability

Coordinates and EM maps have been deposited with the PDB or EMDB: PDB IDs for the crystal structures of Fab17, Fab34, Fab49, Fab34/7-mer peptide and Fab34/18-mer peptide are 9HV1, 9HV2, 9HV8, 9HV9 and 9HVA, respectively. EMDB IDs for the coordinates/EM maps of Fab17/O1M, Fab34/O1M and Fab49/O1M complexes are EMD-54248, EMD-54261 and EMD-54263, respectively. Source data are provided with this paper. Any supplementary information related to this work can be requested to the corresponding authors.
