# Supplementary material for: Cattle antibodies identify a cross-serotype broadly neutralising foot-and-mouth disease virus epitope
Source: NPJ Vaccines. 2026 Apr 2;11:109. doi: 10.1038/s41541-026-01427-7 (PMC13216289; doi:10.1038/s41541-026-01427-7)
Supplement: Supplementary file 1 — Supplementary Information [file 41541_2026_1427_MOESM1_ESM.pdf]

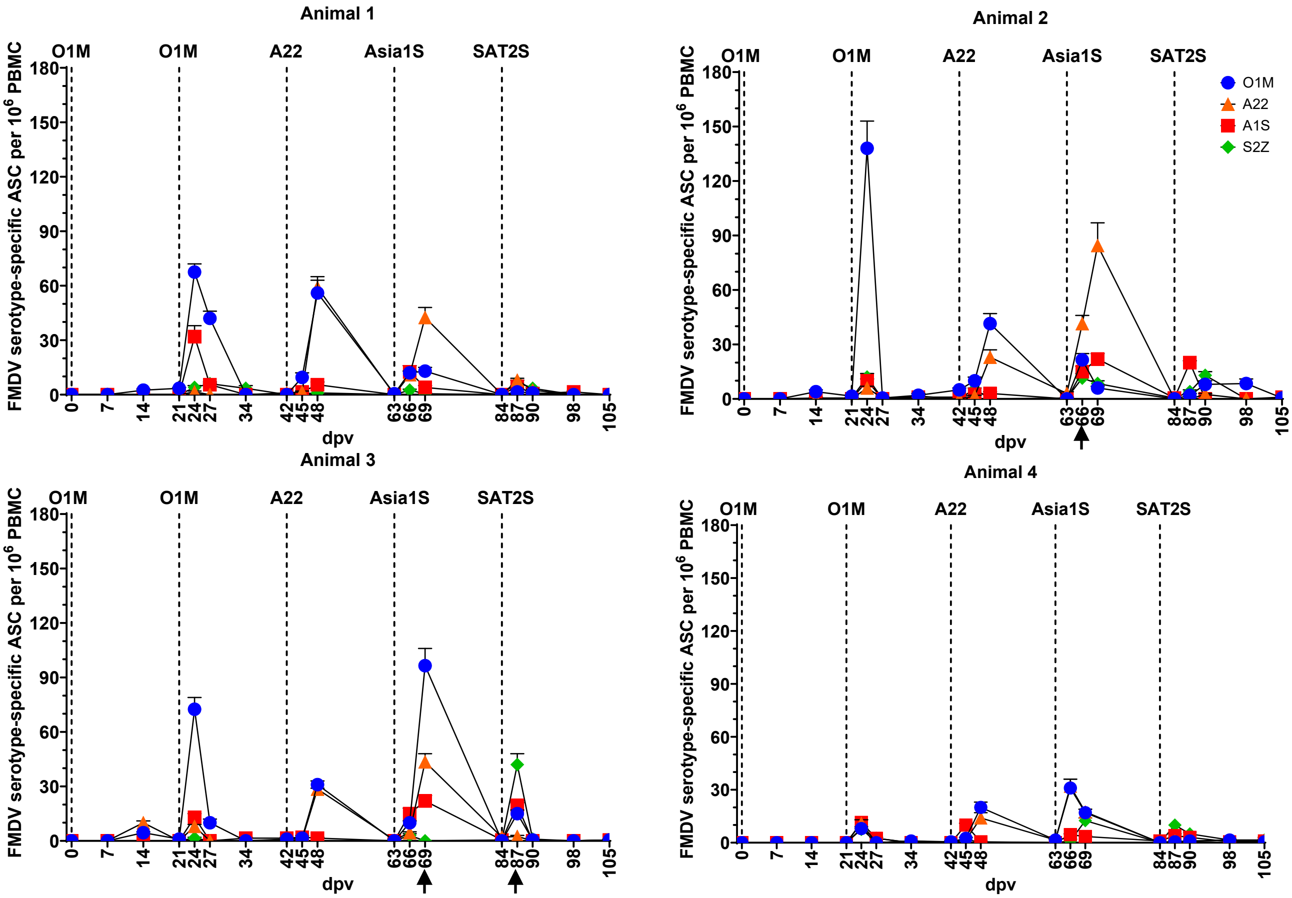

Supplementary Figure 1. Kinetics of the FMDV-specific plasma cell response in individual cattle during the serial heterologous vaccinations with FMDV antigens. Average numbers of FMDV-specific antibody secreting B cells (ASC) per 10^6 PBMCs are shown for each serotype and each sampling day along the 105 days post prime-vaccination (dpv). Vaccination timepoints are indicated by dotted lines. Results are expressed as the duplicate mean + SEM for each animal. Arrows indicate samples (animals and dpv) that were further selected for this study.

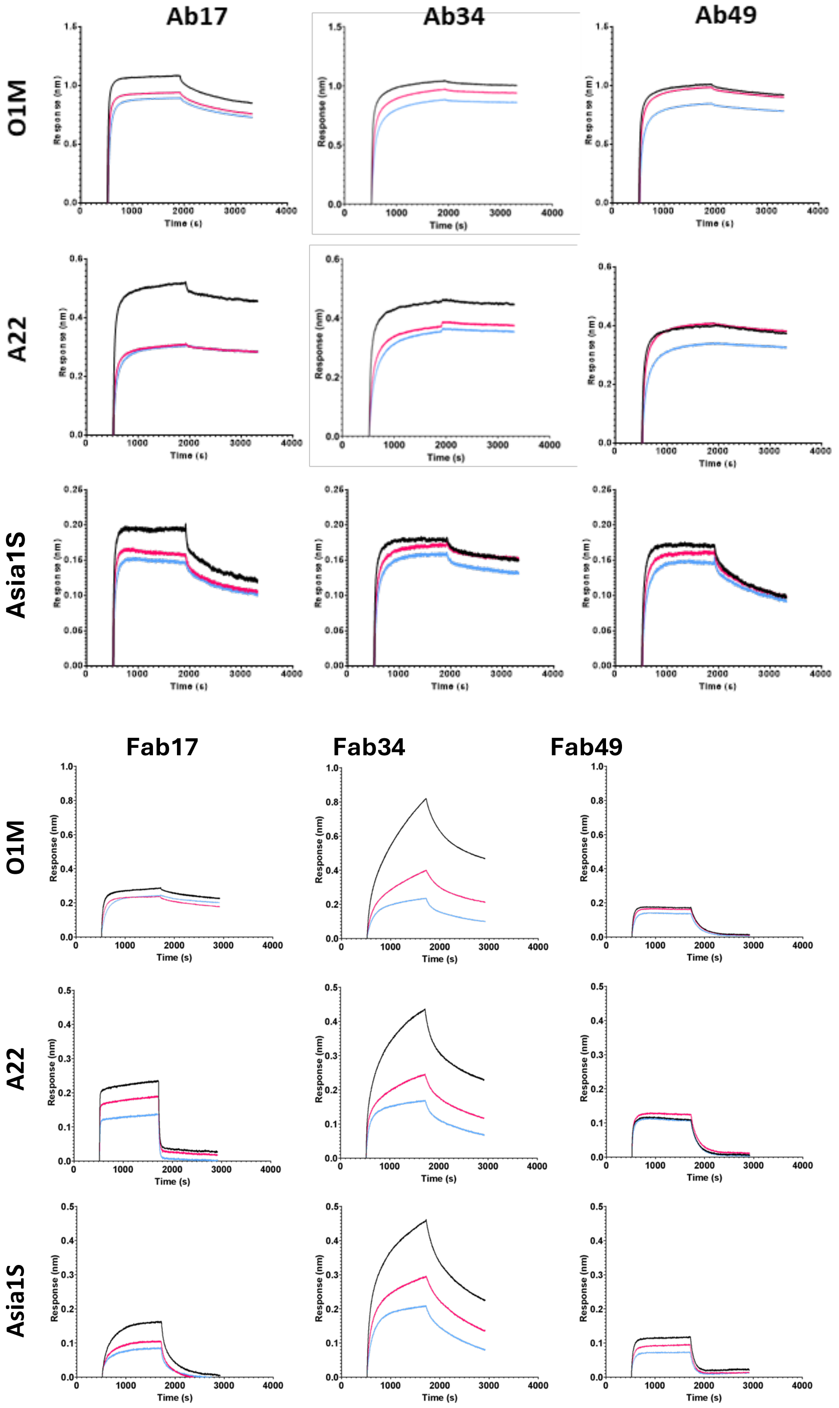

Supplementary Figure 2. Tri-serotype specific Ab and Fab binding kinetics to FMDV VLPs. Representative sensorgrams from two replicates of Ab17, Ab34, and Ab49 (top panel) as well as Fab17, Fab34, and Fab49 (bottom panel) binding to O1M, A22, and Asia1S VLPs. Data are shown for 100 nM (black), 50 nM (pink) and 25 nM (light blue) of Fabs for each serotype.

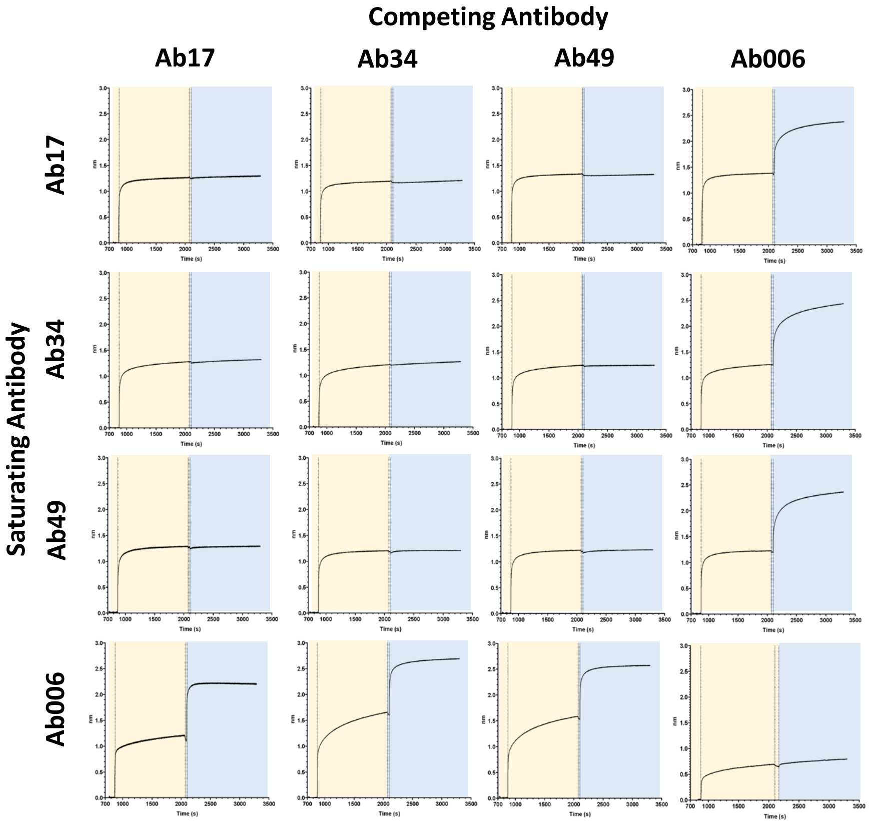

Supplementary Figure 3. The three tri-serotype specific antibodies compete to bind overlapping epitopes. A BLI competition assay was used to measure the responses (in nm) from sequential binding of pairs of antibodies to A22 VLPs. Signal obtained from antibody binding to sensor-bound antigens are indicated within yellow background for primary antibody, or blue background for second antibody. No increase in signal during addition of second antibody indicates blocking.

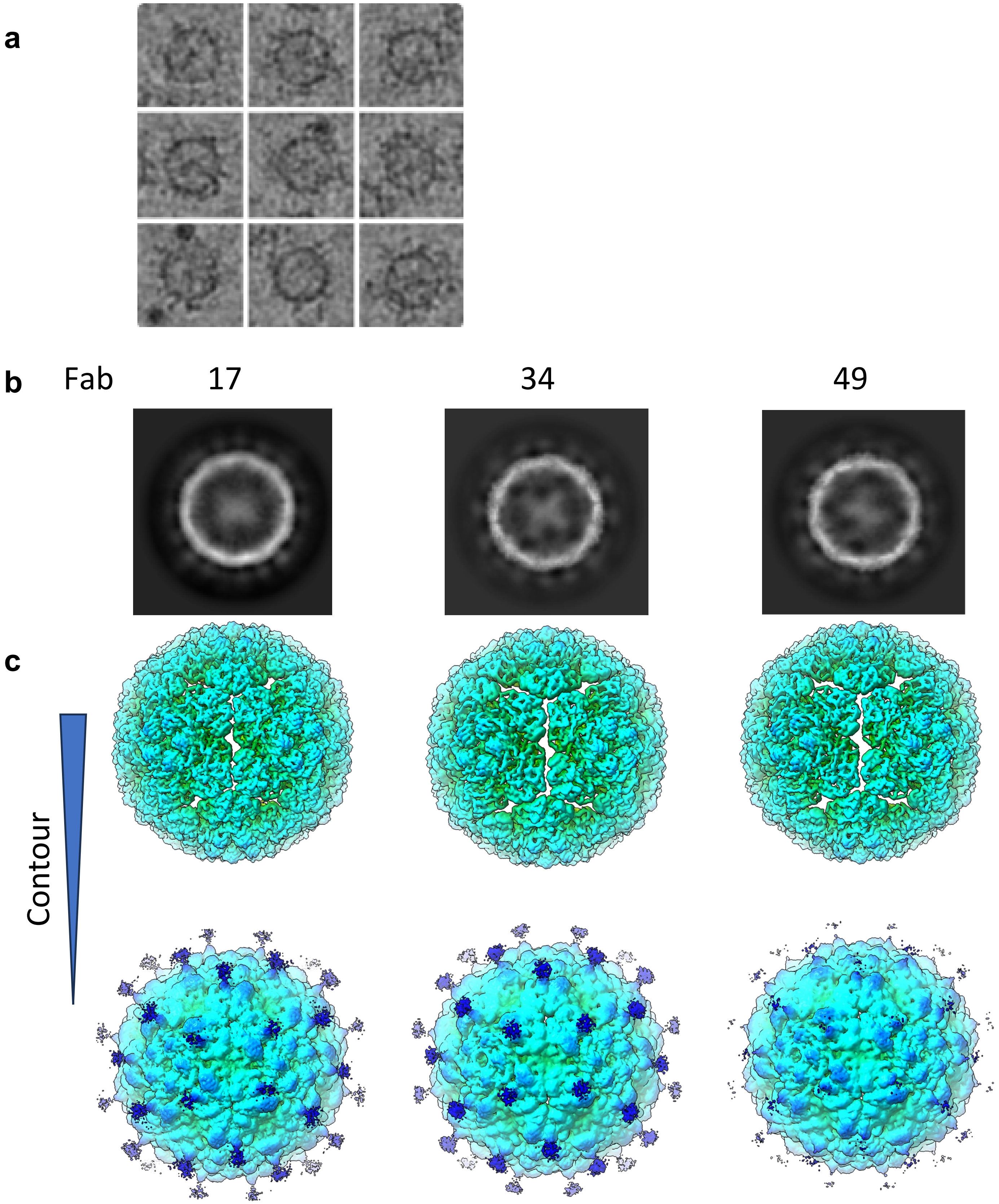

Supplementary Figure 4. **(a)** Example selected particles from O1M-Fab34 complex cryo-EM data showing clear decoration. **(b)** 2D class average for each of the Fab-O1M complexes. **(c)** Globally refined unsharpened maps coloured by diameter (green 280, blue 320 AÅ diameter).

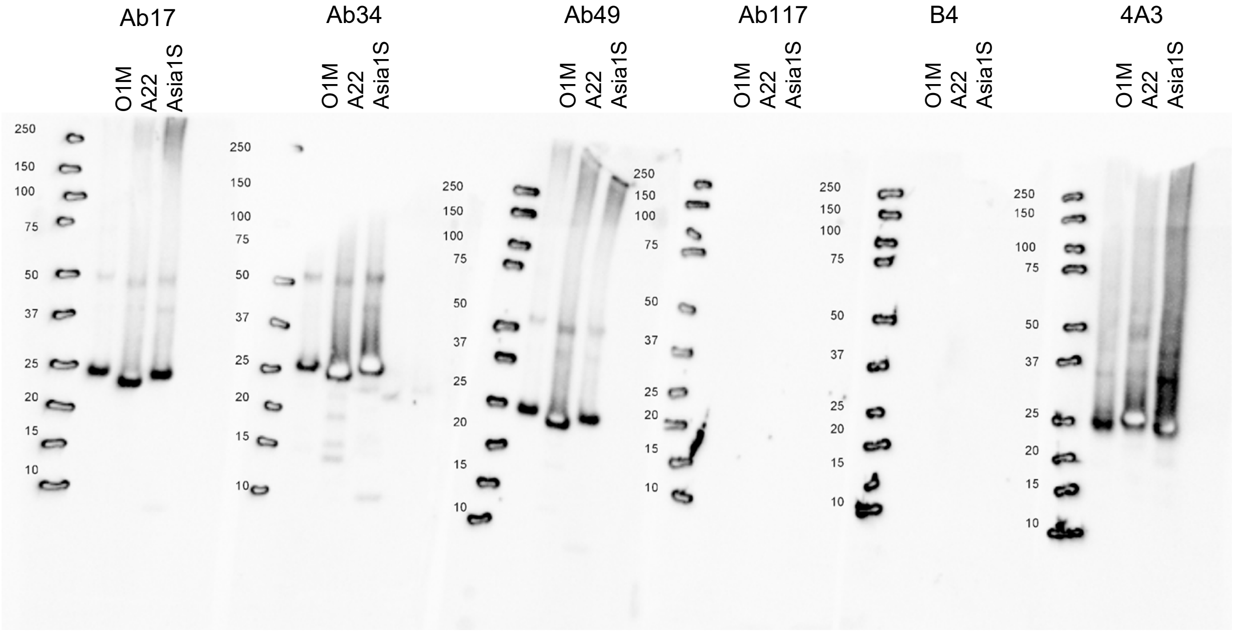

Supplementary Figure 5: **Western blotting indicating the presence of a linear epitope sufficient for tri-specific antibody binding** to a structural protein of the FMDV capsids, O1M, A22 and Asia1S. Ab117 (an antibody ascertained to bind an internal conformational epitope), and B4 (a bovine anti-RSV antibody) show no binding to any of the FMDV capsid proteins. Mouse-anti-VP2 antibody 4A3 (known to bind a linear epitope) serves as a control for binding to a linear epitope. Ab117 blot from Clarke *et al.* [1].

[1] Clarke, J.D., et al., *A broadly reactive ultralong bovine antibody that can determine the integrity of foot-and-mouth disease virus capsids.* J Gen Virol, 2024. 105(10).

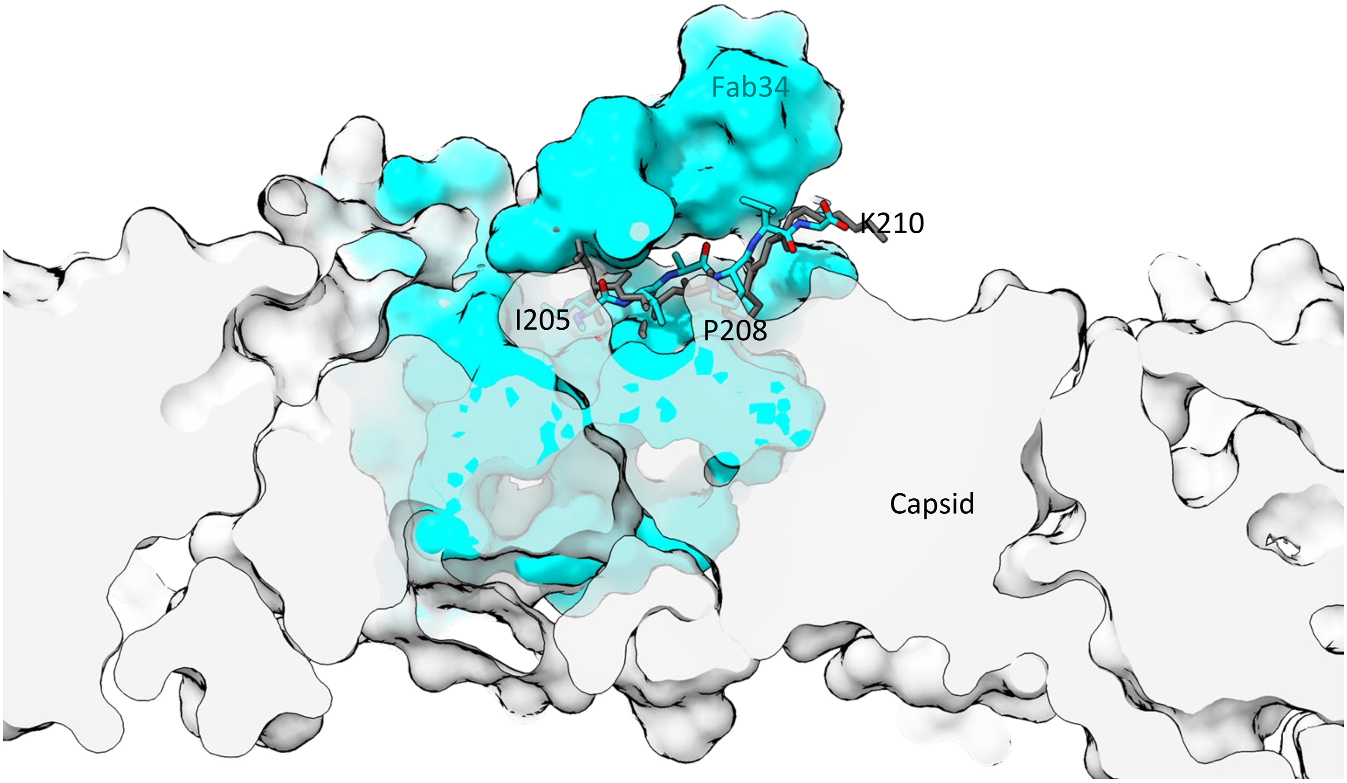

Supplementary Figure 6. VP1 C-terminus must substantially reorder to accommodate Fab binding. The C-terminal peptide regions have been superimposed between PDB:5D8A, where the C-terminal peptide is in dark grey atomic representation with bulk capsid in light grey surface representation and the 7-mer peptide/Fab34 complex both shown in cyan with the Fab is shown in surface representation and the peptide in stick representation. Note that the bulk of the Fab overlaps the capsid, requiring the C-terminus to be dislodged and reorientated to allow antibody binding. Alignment was performed using the matchmaker tool in ChimeraX and based on the 7-mer peptide and native C-terminus of VP1.

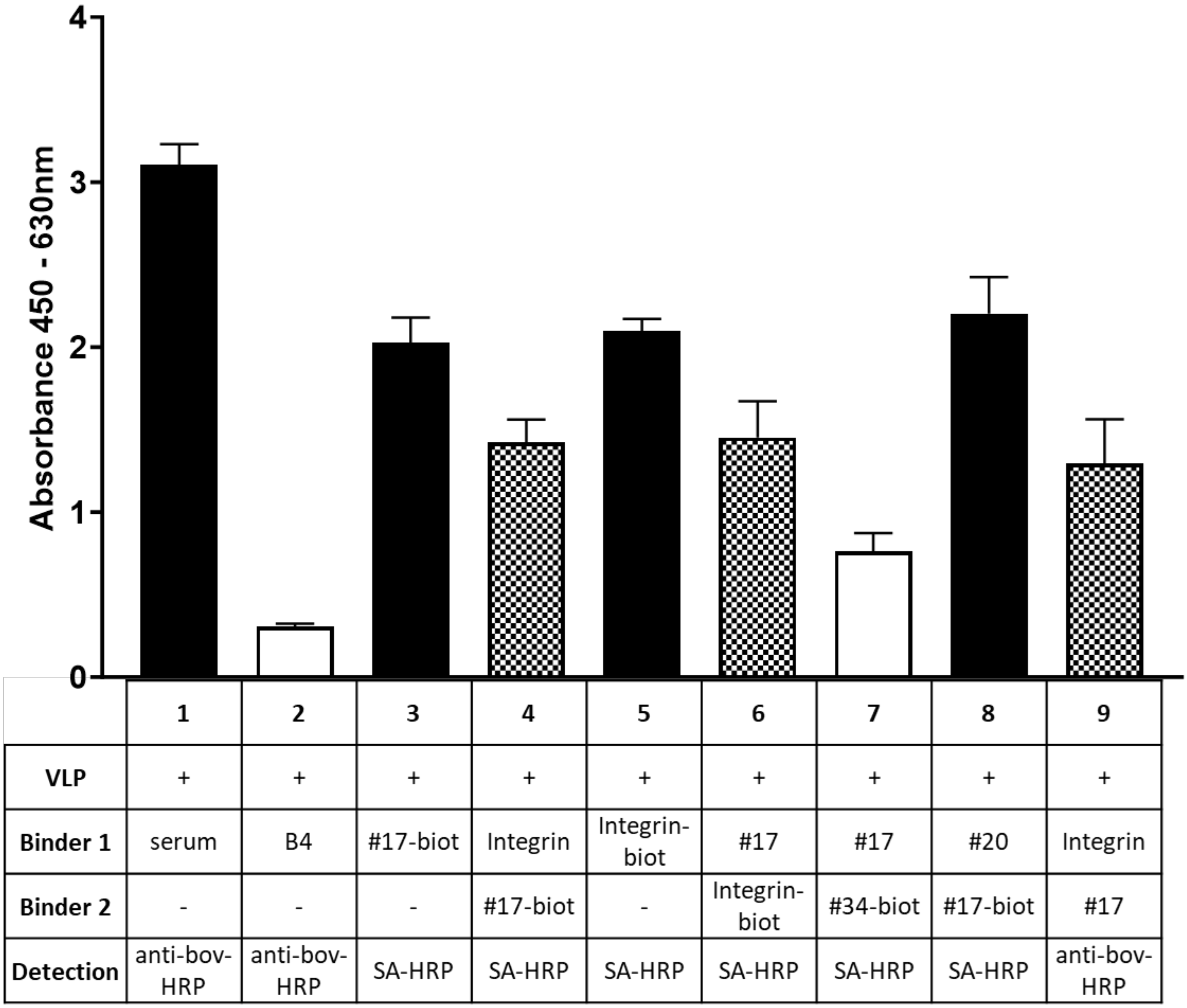

	1	2	3	4	5	6	7	8	9
**VLP**	+	+	+	+	+	+	+	+	+
**Binder 1**	serum	B4	#17-biot	Integrin	Integrin-biot	#17	#17	#20	Integrin
**Binder 2**	-	-	-	#17-biot	-	Integrin-biot	#34-biot	#17-biot	#17
**Detection**	anti-bov-HRP	anti-bov-HRP	SA-HRP	SA-HRP	SA-HRP	SA-HRP	SA-HRP	SA-HRP	anti-bov-HRP

Supplementary Figure 7. Integrin and Ab17 competition ELISA. Mean absorbance at 450 nm with background subtraction at 630 nm is shown ± SEM (n=2). Black bars indicate known positive controls or permissive interactions. White bars indicate known negative controls or blocking interactions. Hatched bars indicate combinations of integrin and Ab17 tested. A blocking interaction is indicated by a reduction in absorbance vs positive control wells. Serum from FMDV-vaccinated animals was used as a positive control [1]. Antibody #20 was used as a non-competing antibody control. Anti-bov-HRP: anti bovine IgG (H+L) antibody coupled to HRP; SA-HRP: streptavidin coupled to HRP.

[1] Grant, C.F.J., et al., The B Cell Response to Foot-and-Mouth Disease Virus in Cattle following Sequential Vaccination with Multiple Serotypes. J Virol, 2017. 91(9).

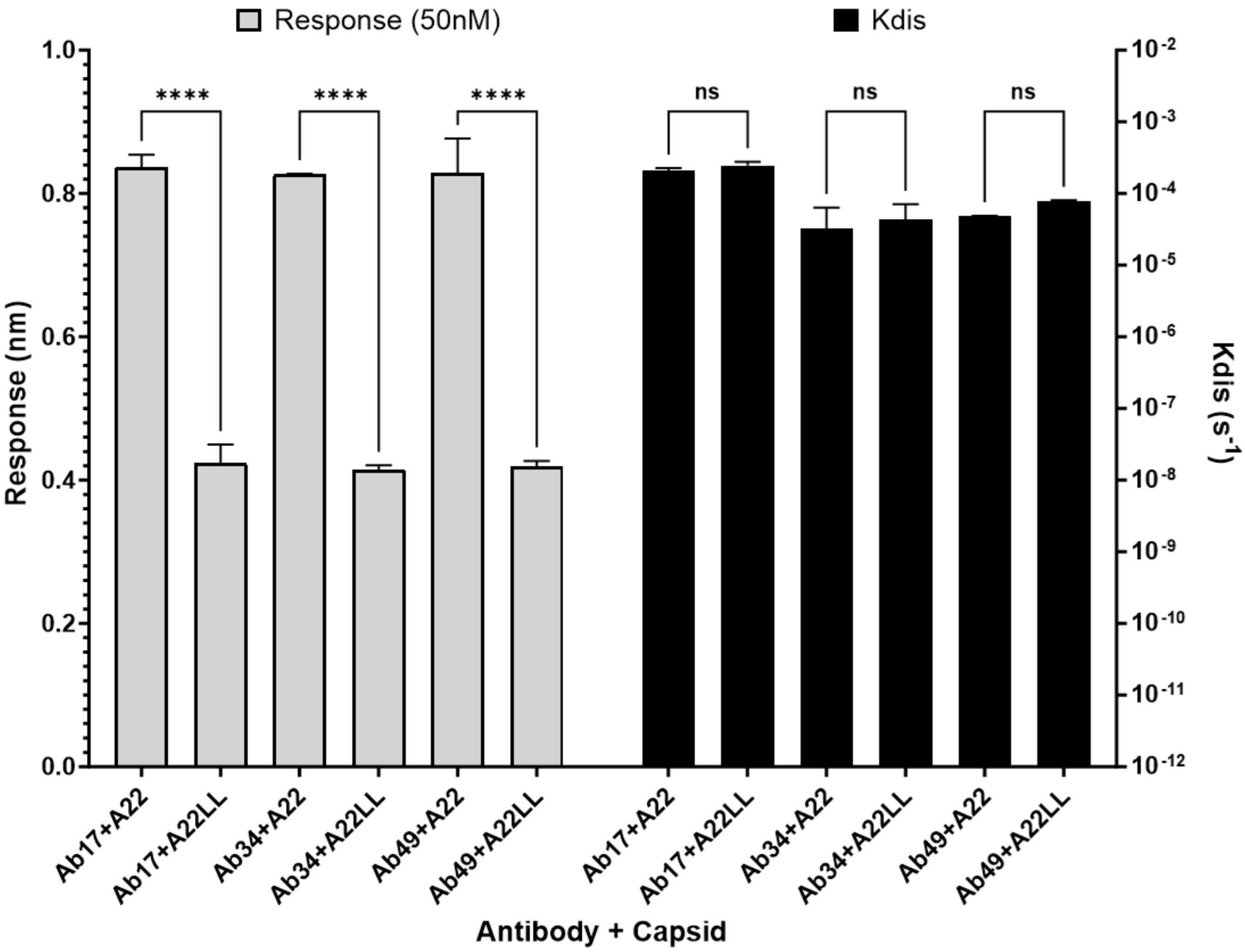

Supplementary Figure 8: Tri-serotype specific antibody binding is reduced in a virus lacking the G-H loop.

Response (grey) and dissociation constant (Kdis, black) from BLI of tri-serotype specific antibodies with standard and G-H loopless (LL) A22 VLPs. Mean of two replicates shown ± SEM. ns = not significant, **** = $p < 0.0001$

Supplementary Table 1: SAT2 topotypes sequence comparison. (a) Percentages of identities of VP1 amino acid sequences between the three SAT2 topotypes used in the study. **(b)** Alignment of VP1 amino acid sequences from the three SAT2 topotypes used in the study.

a

Topotype	VP1 Sequence (GB accession)	SAU/6/2000	ZIM/7/83	ETH/65/2009
VII	SAU/6/2000 (AF367135)	100		
II	ZIM/7/83 (DQ009726)	74.1	100	
XIII	ETH/65/2009 (KF112945)	81.5	77.8	100

b

```
                                    10        20        30        40        50        60        70        80
                           ....|....|....|....|....|....|....|....|....|....|....|....|....|....|....|....|
SAT2/SAU/6/2000 (AF367135) TTSAGESADVVTTDPSTHGGNVQEGRRKHTEVAFLLDRSTHVHTNKTSFVVDLMDTKEKALVGAILRASTYYFCDLEIAC
SAT2/ZIM/7/83 (AF540910)   ...S..G.............A.T.KK.V..D...VM..F...L..R.A.A......N..T...GL...A...........
SAT2/ETH/65/2009 (KF112945) ....S.G......G.A....VANTA..A..D...........Y......SI...E............SA...........

                                    90       100       110       120       130       140       150       160
                           ....|....|....|....|....|....|....|....|....|....|....|....|....|....|....|....|
SAT2/SAU/6/2000 (AF367135) VGDXTRAFWQPNGAPRTTQLGDNPMVFAKGGVTRFAIPFTAPHRLLSTVYNGECVYKKTPTAIRGDRAALAAKYADSTHT
SAT2/ZIM/7/83 (AF540910)   L.EHE.VW..........T.R......SHNN.....V.Y.........R.....K.TQQS........V......NTK.K
SAT2/ETH/65/2009 (KF112945) ..EHK.V...........T.........HN........Y...............T.T..S.P............R.TG.S

                                   170       180       190       200       210
                           ....|....|....|....|....|....|....|....|....|....|....
SAT2/SAU/6/2000 (AF367135) LPSTFNFGFVTVDKPVDVYYRMKRAELYCPRPLLPAYEHTGGDRFDAPIGVERQ
SAT2/ZIM/7/83 (AF540910)   ........H..A.......................G.D.ADR....S.....K.
SAT2/ETH/65/2009 (KF112945) ...........A.........................S.N.R..........K.
```

Supplementary Table 2: Total numbers of FMDV-specific antibody secreting cells per 10^6 PBMCs identified by ELISpot in PBMCs during the sequential vaccination study.

		Vaccine Group (n = 4)				Control Group (n = 2)			
Vaccination	Dpv	O1M	A22	Asia1S	SAT2Z	O1M	A22	Asia1S	SAT2Z
**Prime: O1M**	**0**	0 ± 0	0 ± 0	0 ± 0	0 ± 0	0 ± 0	0 ± 0	0 ± 0	0 ± 0
	7	0 ± 0	0 ± 0	0 ± 0	0 ± 0	0 ± 0	0 ± 0	0 ± 0	0 ± 0
	14	4 ± 0	3 ± 3	0 ± 0	0 ± 0	0 ± 0	0 ± 0	0 ± 0	0 ± 0
**Boost: O1M**	**21**	2 ± 1	0 ± 0	0 ± 0	0 ± 0	0 ± 0	0 ± 0	0 ± 0	0 ± 0
	24	87 ± 22	6 ± 1	28 ± 11	7 ± 3	0 ± 0	0 ± 0	0 ± 0	0 ± 0
	27	15 ±10	0 ± 0	2 ± 1	2 ± 2	0 ± 0	0 ± 0	0 ± 0	0 ± 0
	34	1 ± 1	0 ± 0	0 ± 0	1 ± 1	0 ± 0	0 ± 0	0 ± 0	0 ± 0
**A22**	**42**	2 ± 1	0 ± 0	0 ± 0	0 ± 0	0 ± 0	0 ± 0	0 ± 0	0 ± 0
	45	12 ± 4	2 ± 0	4 ± 2	2 ± 1	0 ± 0	0 ± 0	0 ± 0	0 ± 0
	48	39 ± 11	34 ± 11	3 ± 1	0 ± 0	0 ± 0	0 ± 0	0 ± 0	0 ± 0
**Asia1S**	**63**	0 ± 0	2 ± 1	0 ± 0	0 ± 0	0 ± 0	0 ± 0	0 ± 0	0 ± 0
	66	15 ± 3	25 ± 10	15 ± 5	6 ± 3	0 ± 0	0 ± 0	0 ± 0	0 ± 0
	69	33 ± 24	47 ± 11	14 ± 5	6 ±3	0 ± 0	0 ± 0	0 ± 0	0 ± 0
**SAT2S**	**84**	0 ± 0	1 ± 0	1 ± 0	1 ± 0	0 ± 0	0 ± 0	0 ± 0	0 ± 0
	87	6 ± 4	3 ± 2	11 ± 4	18 ± 10	0 ± 0	0 ± 0	0 ± 0	0 ± 0
	90	4 ± 2	2 ± 1	3 ± 2	7 ± 3	0 ± 0	0 ± 0	0 ± 0	0 ± 0
	98	3 ± 3	1 ± 1	1 ± 0	1 ± 0	0 ± 0	0 ± 0	0 ± 0	0 ± 0
	105	1 ± 0	1 ± 0	1 ± 0	1 ± 1	0 ± 0	0 ± 0	0 ± 0	0 ± 0

Supplementary Table 3: Table summarising the origin (timepoint and animal), closest germline V gene segment and percentage identity to germline for each of the 24 binder antibodies

Antibody ID	Animal	Timepoint (dpv)	Heavy Chain		Light Chain	
			Closest VH gene segment	% Identity to germline	Closest VL gene segment	% Identity to germline
Ab2	2	66	IGHV1-17	93.1	IGLV1-67	93.2
Ab6	2	66	IGHV1-21	92.1	IGLV1-67	98.6
Ab12	3	69	IGHV1-20	94.6	IGLV1-67	95.6
Ab14	3	69	IGHV1-10	97.2	IGLV1-67	96.3
Ab17	3	69	IGHV1-27	93.4	IGLV3-4	98.2
Ab20	3	69	IGHV1-10	93.8	IGLV1-67	95.9
Ab21	3	69	IGHV1-21	94.1	IGLV1-21	92.2
Ab25	3	69	IGHV1-10	95.9	IGLV1-67	96.3
Ab27	2	66	IGHV1-20	94.5	IGLV3-4	96.4
Ab30	2	66	IGHV1-7	93.6	IGLV1-47	99.3
Ab34	3	69	IGHV1-14	92.4	IGLV3-4	96.9
Ab42	3	69	IGHV1-10	97.2	IGLV1-67	97.3
Ab48	3	69	IGHV1-20	92	IGLV1-21	95.6
Ab49	3	69	IGHV1-20	91	IGLV3-4	97.2
Ab69	3	87	IGHV1-21	94.8	IGLV1-67	93.9
Ab74	3	87	IGHV1-20	93.5	IGLV1-67	95.6
Ab88	3	87	IGHV1-20	92.4	IGLV1-21	95.6
Ab94	3	87	IGHV1-14	94.2	IGLV1-21	95.9
Ab97	3	87	IGHV1-17	91.8	IGLV1-67	94.8
Ab108	3	87	IGHV1-20	89.3	IGLV1-67	94.5
Ab112	3	87	IGHV1-17	90.1	IGLV1-67	95.6
Ab122	3	87	IGHV1-17	94.8	IGLV1-67	95.3
Ab125	3	87	IGHV1-10	95.2	IGLV1-67	94.9
Ab139	3	87	IGHV1-21	92.7	IGLV1-47	97.6

Supplementary Table 4: Binding kinetics data from BLI analysis and VNTs of the three tri-serotype specific IgGs to O1M, A22, and Asia1S VLPs. The average BLI response at 50 nM, association rate (Ka), dissociation rate (Kdis), and equilibrium dissociation constant (KD) are shown for each antibody. The average minimum antibody concentration (Min' [ ], in nM) to achieve neutralisation of each serotype from 3 independent experiments with SEM is also shown.

		Response (nm)		KD (M)		Ka (Ms^{-1})		Kdis (s^{-1})		Neutralisation	
		Average	SEM	Average	SEM	Average	SEM	Average	SEM	Min' [ ] (nM)	SEM
Ab17	O1M	1.46	0.1	2.10E-11	6.59E-12	3.03E+05	6.80E+04	7.96E-06	1.63E-06	3.99	2.75
	A22	0.7	0.12	2.46E-10	1.06E-11	6.85E+05	8.31E+03	1.64E-04	5.17E-06	5.49	3.84
	Asia1S	0.19	0.02	2.72E-10	5.35E-11	6.29E+05	2.38E+04	1.97E-04	3.94E-05	63.30	44.01
Ab34	O1M	1.39	0.03	9.04E-12	8.95E-13	2.03E+05	2.99E+04	7.23E-07	6.07E-07	5.49	3.84
	A22	0.57	0.19	9.30E-12	5.53E-12	4.20E+05	6.19E+04	3.19E-06	3.08E-06	5.77	0.96
	Asia1S	0.22	0.02	2.28E-11	6.43E-12	4.36E+05	1.15E+05	7.56E-06	7.45E-06	18.26	8.58
Ab49	O1M	0.93	0.26	1.56E-10	8.34E-11	2.57E+05	2.77E+04	4.90E-05	1.14E-05	5.84	3.43
	A22	0.61	0.19	1.14E-10	1.42E-11	3.89E+05	3.30E+04	3.61E-05	1.21E-05	4.19	2.54
	Asia1S	0.19	0.02	1.11E-09	1.17E-10	2.67E+05	1.43E+04	3.01E-04	3.05E-05	56.30	17.64

Supplementary Table 5: Broad neutralisation by Ab17, 34, and 49 of FMDV serotypes. Summary of the FMDV isolates used by the World reference laboratory FMD (WRLFMD) to test for broad neutralisation of the tri-serotype specific antibodies using VNT. Vaccine strains are highlighted in blue text.

WRLFMD Ref. No.	Country	Serotype	Topotype	Lineage
KEN/8/2021	Kenya	O	EA-2	-
COD/82/2021	DRC	O	EA-2	-
ZAM/11/2021	Zambia	O	EA-2	-
ETH/26/2018	Ethiopia	O	EA-3	-
CIV/3/2018	Côte d'Ivoire	O	EA-3	-
GNA/3/2018	Guinea	O	EA-3	-
ETH/14/2019	Ethiopia	O	EA-4	-
ETH/37/2018	Ethiopia	O	EA-3	-
ZAM/8/2019	Zambia	O	EA-2	-
ERI/8/2017	Eritrea	O	EA-3	-
Manisa/TUR/69	Turkey	O	ME-SA	-
ETH/37/2018	Ethiopia	A	AFRICA	G-IV
KEN/10/2021	Kenya	A	AFRICA	G-I
ETH/70/2018	Ethiopia	A	AFRICA	G-IV
UGA/42/2019	Uganda	A	AFRICA	G-I
ETH/19/2019	Ethiopia	A	AFRICA	G-IV
ERI/7/2018	Eritrea	A	AFRICA	G-IV
IRQ/24/64	Iraq	A	ASIA	A_{22}
PAK/75/2019	Pakistan	Asia 1	ASIA	Sindh-08
MYA/3/2017	Myanmar	Asia 1	ASIA	G-VIII
NEP/46/2017	Nepal	Asia 1	ASIA	G-IX
Shamir/ISR/89	Israel	Asia 1	ASIA	-
KEN/9/2020	Kenya	SAT 1	I	-
MAL/1/2016	Malawi	SAT 1	I	-
BOT/8/2015	Botswana	SAT 1	III	-
SAU/6/2000	Saudi Arabia	SAT 2	VII	-
ZAM/8/2021	Zambia	SAT 2	IV	-
ZAM/12/2019	Zambia	SAT 2	I	-
ERI/29/2019	Eritrea	SAT 2	VII	Lib-12
NIG/8/2020	Nigeria	SAT 2	VII	Lib-12
ZAM/9/2018	Zambia	SAT 3	II	-
ZAM/3/2015	Zambia	SAT 3	II	-
UGA/10/97	Uganda	SAT 3	V	-
Oberbayern/GER/60	Germany	C	EURO-SA	C_1
KEN/1/2004	Kenya	C	AFRICA	-
NEP/35/1996	Nepal	C	ASIA	-
PHI/3/1994	Philippines	C	EURO-SA	C_3R

Supplementary Table 6: Broad binding of Ab17, 34, and 49 to FMDV serotypes. Summary of the WRLFMD FMDV isolates used to test for broad binding of the tri-serotype specific antibodies by ELISA. Vaccination strains are highlighted in blue.

Serotype	Country	Topotype	Lineage	Virus Strain
O	Iran	ME-SA	Ind-2001/d	IRN/72/2009
O	Nepal	ME-SA	Ind-2001/e	NEP/17/2016
O	Kuwait	ME-SA	Ind-2001/a	KUW/3/1997
O	Oman	ME-SA	Ind-2001/b	OMN/7/2001
O	Palestinian Authority	ME-SA	PanAsia	PAT/6/2015
O	Bangladesh	ME-SA		BAN/5/2009
O	Bahrain	ME-SA	PanAsia-2/ANT-10	BAR/1/2014
O	Pakistan	ME-SA	PanAsia-2/ANT-10	PAK/16/2010
O	Iran	ME-SA	PanAsia-2/FAR-09	IRN/29/2013
O	Iran	ME-SA	PanAsia-2/BAL-09	IRN/18/2010
O	Kuwait	ME-SA	PanAsia2/QOM-15	KUW/1/2016
O	Egypt	ME-SA		EGY/32/2009
**O**	**Turkey**	**ME-SA**		**Manisa/TUR/69**
O	Malaysia	SEA		MAY/3/2014
O	Myanmar	SEA	Mya-98	MYA/5/2015
O	Nigeria	WA		NIG/3/2014
O	Egypt	EA-3		EGY/36/2014
O	Egypt	EA-3		EGY/18/2016
O	Tanzania	EA-2		TAN/4/2014
O	Hong Kong	CATHAY		HKN/1/2015
C	Kenya	Africa		KEN/1/2004
C	Nepal	Asia		NEP/35/1996
C	Philippines	EURO-SA	C3R	PHI/3/1994
A	Afghanistan	ASIA	Iran-05/AFG-07	AFG/6/2007
A	Iran	ASIA	Iran-05	IRN/1/2005
A	Iran	ASIA	Iran-05/FAR-09	IRN/78/2009
A	Egypt	ASIA	Iran-05/BAR-08	EGY/31/2014
A	Pakistan	ASIA	Iran-05/FAR-11	PAK/56/2015
A	Iran	ASIA	Iran-05/SIS-10	IRN/6/2016
**A**	**Iraq**	**ASIA**	**A22**	**IRQ/24/64**
A	Iran	ASIA	G-VII	IRN/1/2016
A	Saudi Arabia	ASIA	G-VII	SAU/15/2016
A	Cambodia	ASIA	Sea-97	CAM/5/2015
A	Myanmar	ASIA	Sea-97	MYA/3/2015
A	Egypt	AFRICA	G-II	EGY/1/1972
A	Kenya	AFRICA	G-III	KEN/3/1964
A	Ethiopia	AFRICA	G-VII	ETH/12/2009
A	Zambia	AFRICA	G-I	ZAM/1/2015
A	Mali	AFRICA	G-VI	MAI/16/2006
A	Egypt	AFRICA	G-VII	EGY/3/2016
Asia	Iran	ASIA	G-VIb	IRN/10/2004
**Asia**	**Israel**	**ASIA**		**ISR/89**
Asia	Indonesia	ASIA	G-V	IND/18/1980
Asia	North Korea	ASIA		NKR/2/2007
Asia	Afghanistan	ASIA	G-I	AFG/1/2001
Asia	Iran	ASIA	Sindh-08	IRN/26/2016
SAT3	Zimbabwe	I (SEZ)		RHO/5/1975
SAT3	South Africa	I (SEZ)		SAR/1/2006
SAT3	South Africa	I (SEZ)		KNP/48/1991
SAT3	Zimbabwe	III (NWZ)		ZIM/P25/1991 (UR-7)
SAT3	Zambia	IV		ZAM/P2/1996 (MUL-4)
SAT3	Botswana	II (WZ)		BOT/P10/2010
SAT3	Zimbabwe	II (WZ)		ZIM/2/1984
SAT3	Zambia	II		ZAM/3/2015
SAT3	Uganda	V		UGA/10/1997
SAT1	Kenya	I (NWZ)		KEN/4/2013
SAT1	Mozambique	I		MOZ/1/1975
SAT1	Zimbabwe	II		ZIM/14/2015
SAT1	Namibia	III (WZ)		NMB/1/2015
SAT1	Uganda	IV (EA-1)		UGA/7/1999
SAT1	Ethiopia	IX		ETH/3/2007
SAT1	Nigeria	V		NIG/1/1976
SAT1	Nigeria	VI		NIG/3/1980
SAT1	Uganda	VII (EA-2)		UGA/47/1971
SAT2	Zambia	IV		ZAM/2/2015
SAT2	Mozambique	I		MOZ/3/2015
SAT2	Botswana	III		BOT/3/2015
SAT2	Ghana	V		GHA/8/1991
SAT2	Zimbabwe	II		ZIM/25/2015
SAT2	Uganda	XII		UGA/3/1976
SAT2	Uganda	XII		UGA/9/1995
SAT2	Uganda	X		UGA/2/2002
SAT2	Sudan	XIII		SUD/6/1977
SAT2	Oman	VII	Alx-12	OMN/3/2015
SAT2	Egypt	VII	Ghb-12	EGY/44/2012
SAT2	Mauritania	VII		MAU/1/2014

Supplementary Table 7: Broad binding of Ab17, 34, and 49 to FMDV serotypes by VNT and ELISA. ELISA and VNT data for all 104 FMDV isolates tested to demonstrate broad binding capability of Ab17, Ab34, and Ab49. Vaccination strains are highlighted in blue. Values shown are the ratio of antibody to integrin binding for each virus strain as calculated by dividing antibody absorbance by integrin absorbance. Table continues on next page

Sero-type	Virus Strain	ELISA						VNT		
		Ab17		Ab34		Ab49		Ab17	Ab34	Ab49
		Mean	SD	Mean	SD	Mean	SD			
O	IRN/72/2009	1.094	0.124	1.171	0.106	1.211	0.096			
O	NEP/17/2016	0.791	0.086	0.869	0.118	0.811	0.102			
O	KUW/3/1997	0.736	0.238	0.828	0.337	0.873	0.377			
O	OMN/7/2001	0.749	0.095	0.797	0.13	0.829	0.171			
O	PAT/6/2015	0.882	0.296	0.942	0.25	0.995	0.323			
O	BAN/5/2009	0.811	0.339	0.884	0.361	0.926	0.394			
O	BAR/1/2014	0.804	0.085	0.863	0.111	0.958	0.155			
O	PAK/16/2010	0.772	0.439	0.645	0.295	0.724	0.358			
O	IRN/29/2013	0.658	0.11	0.763	0.136	0.729	0.179			
O	IRN/18/2010	0.633	0.183	0.822	0.238	0.815	0.259			
O	KUW/1/2016	0.749	0.242	0.844	0.344	0.887	0.375			
O	EGY/32/2009	0.779	0.168	0.816	0.196	0.837	0.168			
**O**	**Manisa/TUR/69**	**1.608**	**0.036**	**1.5**	**0.002**	**1.422**	**0.047**	**3.982**	**5.492**	**5.836**
O	MAY/3/2014	0.701	0.135	0.76	0.167	0.784	0.144			
O	MYA/5/2015	0.803	0.248	0.937	0.355	0.894	0.328			
O	NIG/3/2014	0.863	0.423	0.859	0.399	0.926	0.433			
O	EGY/36/2014	0.877	0.212	0.917	0.235	0.98	0.265			
O	CIV/3/2018							7.621	15.585	21.490
O	GNA/3/2018							3.844	10.710	15.585
O	EGY/18/2016	0.772	0.102	0.828	0.174	0.851	0.165			
O	ERI/8/2017							5.355	21.490	15.585
O	ETH/26/2018							42.911	114.450	171.642
O	ETH/37/2018							31.238	171.642	85.821
O	COD/82/2021							3.844	3.844	7.621
O	KEN/8/2021							1.373	1.923	0.961
O	TAN/4/2014	0.791	0.251	0.866	0.264	0.926	0.284			
O	ZAM/11/2021							0.961	1.923	0.961
O	ZAM/8/2019							0.137	2.678	2.678
O	ETH/14/2019							15.585	15.585	10.710
O	HKN/1/2015	0.693	0.08	0.876	0.162	0.881	0.135			
C	KEN/1/2004	0.083	0.042	0.035	0.014	0.048	0.004	>1373	>1373	>1373
C	NEP/35/1996	0.465	0.003	0.352	0.02	0.138	0.022	>1373	>1373	>1373
C	Oberbayern BHK1 29.06.07							30.569	24.837	96.549
C	PHI/3/1994	0.834	0.03	0.89	0.103	0.963	0.105	212.836	425.671	1146.224
A	AFG/6/2007	1.05	0.46	1.108	0.511	1.281	0.536			
A	IRN/1/2005	1.106	0.006	1.131	0.076	1.413	0.036			
A	IRN/78/2009	0.739	0.006	0.833	0.053	0.872	0.062			
A	EGY/31/2014	0.914	0.067	0.949	0.091	1.002	0.047			
A	PAK/56/2015	0.641	0.353	0.75	0.331	0.84	0.523			
A	IRN/6/2016	0.861	0.054	1.117	0.033	1.078	0.071			
**A**	**IRQ/24/64 (A22)**	**2.357**	**0.047**	**2.288**	**0.022**	**2.453**	**0.012**	**5.492**	**5.767**	**4.188**
A	IRN/1/2016	0.903	0.013	1.022	0.089	1.145	0.035			
A	SAU/15/2016	1.005	0.128	1.032	0.184	1.21	0.056			
A	CAM/5/2015	0.724	0.036	0.841	0.018	0.956	0.11			
A	MYA/3/2015	0.786	0.103	0.859	0.089	0.895	0.079			
A	EGY/1/1972	0.928	0.131	1.18	0.152	1.364	0.181			
A	KEN/3/1964	0.802	0.011	0.888	0.006	0.928	0.071			
A	ETH/12/2009	0.865	0.064	0.965	0.061	0.997	0.122			
A	KEN/10/2021							8.033	22.863	22.863
A	UGA/42/2019							1.991	5.767	8.033

Sero-type	Virus Strain	ELISA						VNT		
		Ab17		Ab34		Ab49				
		Mean	SD	Mean	SD	Mean	SD	Ab17	Ab34	Ab49
A	ZAM/1/2015	0.906	0.076	0.971	0.09	1.144	0.081			
A	MAI/16/2006	0.877	0.074	0.956	0.099	0.99	0.038			
A	EGY/3/2016	0.865	0.03	0.988	0.067	1.049	0.086			
A	ERI/7/2018							4.050	8.033	8.033
A	ETH/37/2018							64.400	93.647	93.647
A	ETH/19/2019							22.863	93.647	64.400
A	ETH/70/2018							5.767	11.465	8.033
Asia	IRN/10/2004	0.841	0.067	0.925	0.075	0.913	0.093			
Asia	MYA/3/2017							42.911	42.911	228.833
Asia	NEP/46/2017							5.355	7.690	15.242
**Asia**	**ISR/89**	**2.092**	**0.026**	**2.087**	**0.041**	**1.957**	**0.082**	**63.302**	**18.262**	**56.299**
Asia	IND/18/1980	0.799	0.085	0.912	0.142	0.861	0.176			
Asia	NKR/2/2007	0.758	0.044	0.784	0.112	0.839	0.093			
Asia	AFG/1/2001	0.826	0.098	0.939	0.099	0.897	0.125			
Asia	IRN/26/2016	0.647	0.121	0.778	0.152	0.704	0.184			
Asia	PAK/75/2019							10.710	30.484	85.821
SAT3	RHO/5/1975	0.097	0.007	0.078	0.018	0.037	0.006			
SAT3	SAR/1/2006	0.054	0.008	0.105	0.038	0.043	0.027			
SAT3	KNP/48/1991	0.263	0.022	0.051	0.013	0.076	0.045			
SAT3	ZIM/P25/1991 (UR-7)	0.026	0.025	0.039	0.026	0.053	0.025			
SAT3	ZAM/P2/1996 (MUL4)	0.026	0.019	0.031	0.026	0.058	0.029			
SAT3	BOT/P10/2010	0.011	0.012	0.045	0.046	0.035	0.019			
SAT3	ZIM/2/1984	0.025	0.023	0.031	0.036	0.044	0.029			
SAT3	ZAM/3/2015	0.035	0.013	0.059	0.045	0.06	0.021	>1373	>1373	>1373
SAT 3	ZAM/9/2018							>1373	>1373	>1373
SAT3	UGA/10/1997	0.023	0.018	0.023	0.019	0.042	0.02	>1373	>1373	>1373
SAT1	KEN/4/2013	0.007	0	0.017	0.006	0.021	0.003	>1373	>1373	>1373
SAT 1	KEN/9/2020							>1373	>1373	>1373
SAT 1	MAL/1/2016							>1373	>1373	>1373
SAT1	MOZ/1/1975	0.014	0.001	0.016	0.028	0.036	0.02			
SAT1	ZIM/14/2015	0.031	0.014	0.039	0.028	0.043	0.015			
SAT 1	BOT/8/2015							>1373	>1373	>1373
SAT1	NMB/1/2015	0.023	0.021	0.045	0.034	0.049	0.014			
SAT1	UGA/7/1999	0.014	0.01	0.05	0.04	0.042	0.006			
SAT1	ETH/3/2007	0.07	0.017	0.186	0.071	0.053	0.015			
SAT1	NIG/1/1976	0.06	0.03	0.079	0.02	0.057	0.002			
SAT1	NIG/3/1980	0.04	0.007	0.048	0.031	0.066	0.022			
SAT1	UGA/47/1971	0.025	0.023	0.02	0.001	0.056	0.004			
SAT2	ZAM/2/2015	0.012	0.001	0.055	0.056	0.038	0.009			
SAT 2	ZAM/8/2021							>1373	>1373	>1373
SAT2	MOZ/3/2015	0.024	0.004	0.034	0.021	0.033	0.009			
SAT 2	ZAM/12/2019							>1373	>1373	>1373
SAT2	BOT/3/2015	0.011	0.009	0.073	0.071	0.033	0.004			
SAT2	GHA/8/1991	0.009	0.004	0.048	0.042	0.045	0.021			
SAT2	ZIM/25/2015	0.013	0.002	0.054	0.053	0.034	0.005			
SAT2	UGA/3/1976	0.012	0.011	0.021	0.017	0.048	0.009			
SAT2	UGA/9/1995	0.035	0	0.06	0.055	0.069	0.006			
SAT2	UGA/2/2002	0.007	0	0.033	0.014	0.075	0.002			
SAT2	SUD/6/1977	-0.014	0.034	-0.031	0.044	0.013	0.012			
SAT 2	ERI/29/2019							>1373	>1373	>1373
SAT2	NIG/8/2020							>1373	>1373	>1373
SAT2	OMN/3/2015	0.008	0.015	0.047	0.054	0.055	0.024			
SAT2	EGY/44/2012	0.043	0.003	0.072	0.01	0.078	0.005			
SAT2	MAU/1/2014	0.011	0.005	0.028	0.019	0.052	0.004			

Supplementary Table 8: (a) Cryo-EM data collection, structure determination and refinement statistics, and (b) crystallographic data collection, structure determination and refinement statistics.

[a] Values in parentheses are for the highest-resolution shell.

a

Structure	Fab17 O1M FMDV	Fab34 O1M FMDV	Fab49 O1M FMDV
**EMDB ID**	**EMD-54248**	**EMD-54261**	**EMD-54263**
**Data collection**			
Voltage (kV)	300	300	300
Frames (EER fractions)	50	50	50
Dose rate (e^-/Å^2/s)	9.33	9.47	8.56
Total dose (e^-/Å^2)	50	50	50
Calibrated pixel size (Å)	0.932	0.932	0.932
Defocus (μm)	0.8-2.6	0.8-2.6	0.8-2.6
Movies	9528	9840	9873
Particles (final)	56801	42467	39994
Symmetry	I2	I2	I2
Map resolution (Å)	2.7	3.4	3.4
Sharpening B-factor (Å^2)	93	129	137
**Refinement**			
No. protein atoms	4084	4084	4084
B factors mean (Å^2)	51	66	95
r.m.s. deviations			
Bond lengths (Å)	0.002	0.003	0.002
Bond angles (°)	0.5	0.5	0.5
Clash score	6.7	6.3	5.6
Ramachandran<br>Favoured<br>Allowed<br>Disallowed (%)	 97.9<br>2.2<br>0.0	 97.5<br>2.5<br>0.0	 97.5<br>2.5<br>0.0
Rotamer outlier (%)	0.7	0.7	1.0
d FSC model (0.5)	2.7	3.7	3.7
CC (mask)	0.82	0.77	0.80

b

	**Fab alone**			**Fab + peptide**	
**Structure**	**Fab 17**	**Fab 34**	**Fab 49**	**Fab34/7-mer**	**Fab34/18-mer**
**PDB ID**	**9HV1**	**9HV2**	**9HV8**	**9HV9**	**9HVA**
**Data collection**					
Space group	$P6_1$	$P2_1$	$P2_12_12_1$	C2	C2
**Cell dimensions**					
a, b, c (Å), α,β,γ γ (°)	136.1, 136.1, 79.7<br>90, 90, 120	71.4, 81.5, 74.5<br>90, 116.0, 90	72.0, 78.6, 100.4<br>90, 90, 90	173.3, 40.7, 71.7<br>90, 94.4, 90	302.5, 111.9, 140.6<br>90, 116.0, 90
Resolution (Å)	68–1.96 (1.99–1.96)[a]	64–1.77(1.8–1.77)	62–1.72 (1.75–1.72)	86–1.80 (1.83–1.80)	75–3.89 (3.96–3.89)
R_{merge}	0.138 (---)	0.090 (---)	0.066 (---)	0.088 (---)	0.387 (---)
R_{pim}	0.031 (2.107)	0.037 (1.123)	0.019 (0.873)	0.036 (1.055)	0.154 (1.245)
I/s(I)	10.4 (0.2)	11.5 (0.3)	17.4 (0.3)	9.1 (0.2)	4.5 (0.5)
$CC_{1/2}$	0.999 (0.384)	0.998 (0.307)	1.000 (0.359)	0.996 (0.294)	0.984 (0.242)
Completeness (%)	100 (100)	97.3 (75.1)	92.1 (55.6)	96.0 (68.0)	99.7 (99.3)
Redundancy	21.1 (21.2)	6.5 (4.2)	12.7 (8.6)	6.7 (5.1)	7.2 (7.6)
**Refinement**					
Resolution (Å)	45–1.96	41–1.77	47–1.72	86–1.80	75–3.89
No. reflections	49239/2748	67693/3614	52784/2570	41635/2040	36828/2001
R_{work} / R_{free}	0.216/0.246	0.213/0.237	0.184/0.215	0.197/0.227	0.230/0.278
**No. atoms**					
Protein	3252	6319	3277	3294	19818
Ligand/ion/water	68	510	383	235	
**B factors (Å^2)**					
Protein	76	43	43	53	189
Ligand/ion/water	69	44	48	61	
**r.m.s. deviations**					
Bond lengths (Å)	0.007	0.003	0.008	0.004	0.002
Bond angles (°)	1.0	0.7	0.9	0.7	0.6

Supplementary Table 9: Binding kinetics data from BLI analysis of the three tri-serotype specific antibodies as Fabs to O1M, A22, and Asia1S VLPs. The average BLI response at 50 nM, association rate (Ka), dissociation rate (Kdis), and equilibrium dissociation constant (KD) are shown for each antibody. The average minimum concentration (Min' [ ]) to achieve neutralisation of each serotype from two independent experiments with SEM is shown.

		Response (nm)		KD (M)		Ka (Ms^{-1})		Kdis (s^{-1})		Neutralisation	
		**Average**	**SEM**	**Average**	**SEM**	**Average**	**SEM**	**Average**	**SEM**	**Min' [ ] (nM)**	**SEM**
**Fab17**	**O1M**	0.306	0.069	5.93E-09	5.35E-09	1.19E+06	8.66E+05	1.17E-02	1.15E-02	>2625	N/A
	**A22**	0.1904	0.002	1.96E-08	1.10E-09	1.52E+06	9.04E+03	2.98E-02	1.85E-03	>2625	N/A
	**Asia1S**	0.1146	0.01	2.92E-07	2.38E-07	2.86E+04	1.78E+04	4.10E-03	1.61E-03	>2625	N/A
**Fab34**	**O1M**	0.387	0.01	1.23E-08	9.00E-09	4.64E+05	4.38E+05	1.77E-03	1.23E-03	>2612.5	N/A
	**A22**	0.1943	0.049	9.90E-09	2.07E-09	7.88E+04	2.70E+03	7.86E-04	1.90E-04	>2612.5	N/A
	**Asia1S**	0.4663	0.172	9.43E-09	2.55E-09	8.71E+04	5.57E+03	8.07E-04	1.69E-04	>2612.5	N/A
**Fab49**	**O1M**	0.1665	0.005	6.37E-09	4.57E-09	5.53E+05	1.36E+05	2.90E-03	1.67E-03	>2600	N/A
	**A22**	0.0793	0.045	4.44E-09	2.65E-09	7.49E+05	6.05E+04	3.49E-03	2.25E-03	>2600	N/A
	**Asia1S**	0.1262	0.032	1.39E-08	1.22E-08	4.89E+05	2.00E+05	4.39E-03	3.15E-03	>2600	N/A

Supplementary Table 10: F(ab')2 neutralisation potency. Average minimum concentration (Min' [ ], in nM) of two independent experiments with SEM is shown when applicable. F(ab')2 of antibody 122, a monospecific neutralising antibody of A22, is shown as a positive control.

		Neutralisation	
		**Min' [ ] (nM)**	**SEM**
**F(ab')2 #17**	**O1M**	1674	876
	**A22**	1874	677
	**Asia1S**	>2550	N/A
**F(ab')2 #34**	**O1M**	1034	237
	**A22**	>2550	N/A
	**Asia1S**	>2550	N/A
**F(ab')2 #49**	**O1M**	>2525	N/A
	**A22**	>2525	N/A
	**Asia1S**	>2525	N/A
**F(ab')2 #122**	**A22**	0.4545	N/A

Supplementary Table 11: Vaccine antigens dose along timeline of vaccination. Amount (µg) of classic FMDV antigen or VLP used for the prime and boost vaccinations of the cattle. Control animals received adjuvant only at each vaccination timepoint.

	Purified classic antigen (µg)				Unpurified VLPs (µg)
	Prime O1M	Boost O1M	Boost A22	Boost A1S	Boost S2S
Animal ID	0dpv	21dpv	42dpv	63dpv	84dpv
Vaccinated group					
#01	8	10	10	10	10
#02	8	10	10	10	10
#03	6	10	10	10	10
#04	8	10	10	10	10
Control group					
#09	0	0	0	0	0
#10	0	0	0	0	0
